# Dampening the Signals Transduced through Hedgehog via MicroRNA miR-7 Facilitates Notch-Induced Tumourigenesis

**DOI:** 10.1371/journal.pbio.1001554

**Published:** 2013-05-07

**Authors:** Vanina G. Da Ros, Irene Gutierrez-Perez, Dolors Ferres-Marco, Maria Dominguez

**Affiliations:** Instituto de Neurociencias, CSIC-UMH, Alicante, Spain; Stanford University, United States of America

## Abstract

Fine-tuned Notch and Hedgehog signalling pathways via attenuators and dampers have long been recognized as important mechanisms to ensure the proper size and differentiation of many organs and tissues. This notion is further supported by identification of mutations in these pathways in human cancer cells. However, although it is common that the Notch and Hedgehog pathways influence growth and patterning within the same organ through the establishment of organizing regions, the cross-talk between these two pathways and how the distinct organizing activities are integrated during growth is poorly understood. Here, in an unbiased genetic screen in the *Drosophila melanogaster* eye, we found that tumour-like growth was provoked by cooperation between the microRNA miR-7 and the Notch pathway. Surprisingly, the molecular basis of this cooperation between miR-7 and Notch converged on the silencing of Hedgehog signalling. In mechanistic terms, miR-7 silenced the *interference hedgehog* (ihog) Hedgehog receptor, while Notch repressed expression of the *brother of ihog* (boi) Hedgehog receptor. Tumourigenesis was induced co-operatively following Notch activation and reduced Hedgehog signalling, either via overexpression of the microRNA or through specific down-regulation of *ihog*, *hedgehog*, *smoothened*, or *cubitus interruptus* or via overexpression of the *cubitus interruptus* repressor form. Conversely, increasing Hedgehog signalling prevented eye overgrowth induced by the microRNA and Notch pathway. Further, we show that blocking Hh signal transduction in clones of cells mutant for *smoothened* also enhance the organizing activity and growth by Delta-Notch signalling in the wing primordium. Together, these findings uncover a hitherto unsuspected tumour suppressor role for the Hedgehog signalling and reveal an unanticipated cooperative antagonism between two pathways extensively used in growth control and cancer.

## Abstract 2

Analysis of tumorigenesis in *Drosophila* reveals a tumor-suppressor role for Hedgehog signaling in the context of oncogenic Notch signaling.

## Author Summary

Growth control mechanisms ensure that organs attain the correct final size, generally averting tumour growth. This control is often linked to spatially confined domains known as organizers (conserved signalling centres), established along the dorsal-ventral and anterior-posterior axes of the organ by the Notch and Hedgehog pathways, respectively. The organizers emit signals that dictate growth, cell fate specification, and differentiation. However, how the distinct organizing signals received are integrated by cells within a growing organ remains a mystery. By studying how Delta-Notch signalling drives tumorigenesis, we identified the conserved microRNA miR-7 as a co-operative element in tumorigenesis mediated by Delta. We found that the cooperation between the microRNA and Delta-Notch pathway converged on the silencing of two obligatory and functionally redundant Hedgehog receptors, interference hedgehog and brother of ihog. Downregulation of other *hedgehog* pathway genes via RNA interference or genetic mosaics revealed a tumour suppressor role for Hedgehog signalling in the context of the oncogenic Notch pathway. Given the conservation of miR-7, as well as of the Notch and Hedgehog pathways, the conclusions we have drawn from these studies on *Drosophila* may be applicable to some human cancers.

## Introduction

A fundamental question in biology is what instructs cells to stop growing when the proper size is attained to commence terminal differentiation. Indeed, this issue is relevant not only to size regulation but also to cancer. One strategy that organisms use to promote the growth of organs involves the establishment of spatially confined domains called organizers, conserved signalling centres established along the dorsal-ventral (DV) and anterior-posterior (AP) axes of the organs, often involving members of the Notch (DV organizers) and Hedgehog (Hh) (AP organizers) families. Organizers act as a source of graded signals (e.g., Wingless/Wnts, and BMP/Dpp) that promote global organ growth and subsequently, or concurrently, cell fate specification along the DV or AP axes [1],[2]. Although how individual organizing pathways promote growth has been studied comprehensively (e.g., [3]–[5]), our understanding of how orthogonal organizers are integrated and of the cross-talk between them remains limited. Tumourigenesis may occur if the finely balanced growth-promotion and termination is disrupted. Yet little attention has been paid to the issue of how growth by organizers is terminated.

To discover mechanisms of Notch-induced tumourigenesis in an *in vivo* context, we used the fruit fly *Drosophila melanogaster* compound eye. This tissue provides a particularly powerful tool to define novel oncogenes and tumour suppressor networks via unbiased genome-wide screens. Particularly, the early stages of eye development seem to recapitulate molecular mechanisms in human NOTCH1-induced oncogenesis (e.g., [6]–[10]). Human NOTCH1 can function either as an oncogene or a tumour suppressor depending on the cellular context, which often reflects the physiological role of NOTCH1 in the particular stage or cell type. During early development of the fly eye, the pleiotropic Notch pathway plays a predominant role in growth promotion. Consequently, this tissue and stage is useful to identify contextual factors that may synergize with Notch to foster benign and/or invasive tumour growth *in vivo*.

The growth in the compound eye, which is derived from the centre of the eye imaginal disc, depends on a conserved DV Notch-mediated growth-promoting organizer, which is established early in the second larval instar by the asymmetric activation of the Notch receptor by its ligands Delta and Serrate (DLL1,2,4, and JAG1,2 in humans) along the DV boundary (reviewed in [11]). Downstream of the organizer, *eyegone* (*eyg*) gene is expressed specifically in the organizer cells and it controls global eye size [12],[13]. A similar DV organizer has been found in a variety of contexts, including the fly and vertebrate limbs, although the expression of *eyg* is restricted to the fly eye. Eyg is functionally related to the human PAX6(5a) oncogene [13] and acts as a transcriptional repressor [14],[15] though complementary patterns of expression of the organizer in developing eyes have never been reported.

Growth and retinal differentiation in the eye field is spatially and temporally coordinated. Retinal differentiation depends on a separate organizer, the AP organizer, which is associated with the morphogenetic furrow (MF). The MF begins to form at the posterior margin of the early third instar eye disc, and as it moves in an anterior direction, it leaves differentiated retinal cells in its wake. Just anterior to the MF, eye cells arrest in G1 of the cell cycle prior to the start of differentiation, and most cells then go through a synchronous round of cell division before they terminally exit the cell cycle [16]. The initiation and progression of the MF, and of G1 arrest, is positively regulated by Hh [17]–[24]. Though the initiation and progression of the MF in the developing eye disc follows that of the DV organizer [25], the expression of *hh* gene starts earlier in second instar [19] and hence overlaps in time with the DV growth-promoting organizer (Figure S1). Early studies of ectopic Hh signalling led to the idea that this signal ultimately contributes to retinal patterning and also directly regulate eye growth [18], although more recently it has been shown that when the Hh pathway is constitutively activated (via inactivation of downstream repressors) in cells confined to a clone, the surrounding wild-type cells overproliferate but the cells within the clone show growth disadvantage and eventually are eliminated by apoptosis [26]. The influence of Hh on growth in Notch-mediated growth regulation needs to be investigated by loss-of-function approaches in the appropriate context.

In both flies and humans, Hh signalling relieves the inhibition exerted by Patched (PTCH1 in humans) on the intermediate pathway component Smoothened (Smo/SMO), allowing Smo to stabilize full-length Cubitus interruptus (Ci), which acts as a transcriptional activator (Ci-155: Gli2,3 in mammals) and inhibiting the processing of Ci-155 to the truncated transcriptional repressor (Ci-75, in flies) [27]. In addition to these core components, two related members of the immunoglobulin/fibronectin type III–like superfamily have recently been identified as Hh co-receptors in *Drosophila*, with functionally overlapping roles: Interference hedgehog (Ihog) and Brother of Ihog (Boi) [28]–[32]. Indeed, the human counterparts of these proteins, CDO (named after CAM-related/down-regulated by oncogenes) and BOC (Brother of CDO), also act as obligatory co-receptors for Hh signalling [28],[32]–[41]. While overactive Hh signalling is unreservedly oncogenic, making Hh a prime target for therapeutic interventions, there is evidence that loss-of-function of some components of the Hh pathway may exert a tumour-suppressor role. A notable example is that of CDO and BOC, which were initially isolated on the basis of their downregulation by RAS oncogenes in transformed cells, and that were shown to act as tumour suppressors in vitro [42]. More recently, recurrent somatic mutations in the sonic Hh pathway were identified in human pancreatic cancers through global genomic studies, affecting GLI1, GLI3, and BOC [43]. However, the role of these mutations in cancer remains untested.

Here, we describe the identification of the conserved microRNA (miRNA) miR-7 as a gene that enhances Notch pathway-induced eye overgrowth in *D. melanogaster*. miRNAs are small noncoding RNAs that negatively regulate gene expression by binding to “seed” sequences in the untranslated regions (UTRs) and/or in the open reading frame of target messenger RNAs, thereby inhibiting translation and, at times, indirectly driving mRNA degradation. Although miRNAs are in the front line of cancer research, their role in cancer is often unconfirmed *in vivo*. We identified the *ihog* gene as a functionally relevant, direct target of miR-7 in Notch-mediated tumourigenesis *in vivo*. Further, we provide evidence that the microRNA *mir-*7 and Notch pathway cooperatively dampen Hh signal transduction via down-regulation of its receptors *ihog* and *boi*, respectively. As a consequence, we hypothesize that tumours form by the cooperation between the gain of Dl-Notch signalling and a deficiency to transduce Hh signal. We validated this hypothesis by showing that the inhibition of endogenous Hh core components similarly enhanced Dl-Notch-mediated organizing activity resulting in severe overgrowth both in the eye disc and the wing disc. Conversely, increasing Hh signal transduction pathway suppressed eye tumour-like growth by Dl and the microRNA. Given the conservation of these pathways, similar cooperative antagonistic interactions between oncogenic Notch and loss of Hh signalling might play a role in human cancers.

## Results

### MicroRNA miR-7 Cooperates with Delta to Trigger Severe Overgrowth in *Drosophila* Eye

**Figure 1:**
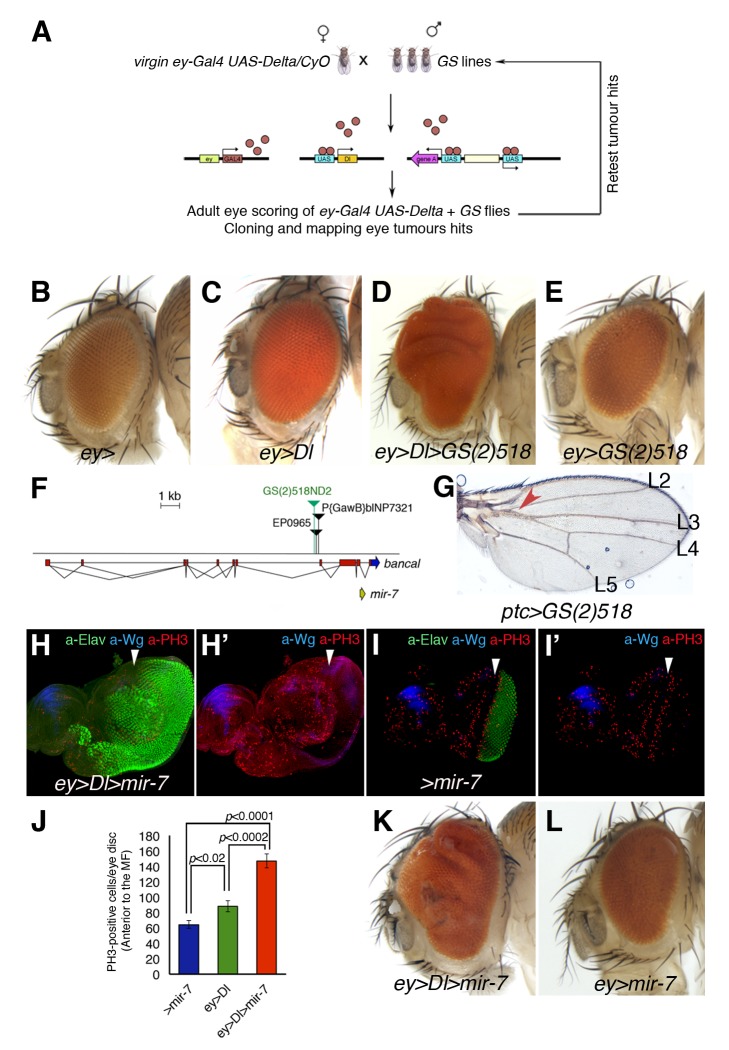
The Conserved MicroRNA miR-7 co-operates with Notch in *D. melanogaster* oncogenesis. (A) A schematic outline of the *Gene Search* (*GS*) gain-of-expression screen for Notch co-operating oncogenes in the developing *Drosophila* eye. (B–E and K–L) Adult heads of control female *ey-Gal4* wild-type eye size (B) and combinations between GS line, UAS transgenes, and *ey-Gal4* are shown. (C) *Dl* expression under the control of *ey-Gal4* results in a mild overgrowth in the eye (130% larger than wild type size). (D) Introducing the *GS(2)518ND2* line enhanced overgrowth by *Dl* (>320%, see also Figure S2). (E) The overexpression of gene(s) affected by the *GS(2)518ND2* line alone causes no overt eye overgrowth. (F) Scheme of the *GS(2)518ND2* insertion. (G) Overexpression of the *GS(2)518ND2* line driven by *ptc-Gal4* showed the typical wing vein L3–L4 fusion. (H–I′) Confocal images of third instar eye-antennal discs stained for the mitotic marker PH3 (red), Wg (blue) to define the DV axis, and the neuronal marker Elav (green) of the indicated genotypes. White arrowheads indicate the position of the MF. The co-expression of *UAS-mir-7* with *UAS-Dl* causes eye disc overgrowth and a front of retinal differentiation highly disorganized (H, compare with control sibling eye disc in I). (J) Quantification of mitotic cells labelled by PH3 anterior to the MF of the genotypes: *ey>Dl>mir-7* (red bar), *ey>Dl* (green bar), and wild-type sibling discs +/*UAS-mir7* (*>mir-7*, blue bar). Data shown represent the mean ± s.e.m. of total PH3 measurement in 20 eye discs per genotype. *P* values were calculated by the unpaired Student's *t* test. (K–L) Adult heads overexpressing *mir-7* driven by *ey-Gal4* in the presence (K) or the absence (L) of the *UAS-Dl* transgene. See also Figures S2 to S4 for supplementary data.

To identify endogenous genetic determinants that may limit Notch-driven tumourigenesis *in vivo*, we carried out an unbiased (genome-wide) gain-of-expression screen for loci that converted Dl-induced mild eye overgrowth into severe overgrowths (benign tumour-like growth: eye tissue is overgrown and folded) or metastatic tumours (provoke secondary eye growths throughout the body). A Gene Search (GS) transposon system was employed to systematically generate gain-of-expression mutations as in [44], using the *eyeless* (*ey*)*-Gal4* to drive expression of UAS-containing transgenes and the GS lines in the imaginal disc cells of the growing eye (the precursors of the adult fly eye; Figure 1A–B). In this way, we identified a *GS* line (*GS(2)518ND2*) that converted *Dl*-induced modest eye overgrowth (Figure 1C; adult eyes are 130% bigger than control wild type eyes) into severely overgrown and folded eye tissue (*ey-Gal4 UAS-Dl GS(2)518ND2*, hereafter *ey>Dl>GS(2)518*) (250%–320% larger than wild-type eyes; 54% penetrance, *n* = 200 eyes; Figure 1D). Differentiation and growth defects of third instar eye discs of *ey>Dl>GS(2)518* are shown in Figure S3. In the absence of *Dl* overexpression, the overexpression or misexpression of the gene(s) affected by *GS(2)518ND2* did not increase eye size (*ey>GS(2)518*; Figure 1E).

The *GS(2)518ND2* line carried an insertion 3.1 kb upstream of the *mir-7* miRNA gene (Figure 1F), which is transcribed from an internal promoter within a 3′ intron of the *bancal*/*heterogeneous nuclear ribonucleoprotein K* (*bl/hnRNP-K*) gene [45]. A set of *EP* elements in the vicinity of *GS(2)518ND2* has been previously described to cause *mir-7* overexpression, and to induce proximal fusion of longitudinal (L) veins 3 and 4, as well as distal wing notching or bristle tufting [45]–[47]. Indeed, expressing *GS(2)518ND2* along the AP compartment boundary in the wing imaginal disc using *patched* (*ptc*)*-Gal4* caused similar L3-–L4 fusion as that reported following *mir-7* overexpression in this domain (*ptc>GS(2)518*; Figure 1G). Conversely, the direct overexpression of *mir-7* together with *Dl* (hereafter, *ey>Dl>mir-7*), using a *mir-7* transgene that does not contain any *bl* sequences (*UAS-mir-7*), provoked overgrown larval eye discs *ey>Dl>mir-7* (Figure 1H; compare with sibling wild type eye discs, Figure 1I) associated with significant increased cell proliferation (Figure 1J and Figure S4C–D,H), resulting in adult overgrown and folded eyes similar to that in the *GS(2)518ND2* flies (70% of adult *ey>Dl>mir-7* animals displayed eye benign tumour-like growth, *n* = 200; Figure 1K and Figure S2A–C). There was no increase in eye size when *UAS-mir-7* alone was overexpressed by *ey-Gal4* (*ey>mir-7*; Figure 1L).

### Identification of Candidate Tumour Suppressor Targets of miR-7 by in Vivo RNAi Screening in the Delta Overexpression Model

In the wing disc, the miR-7 microRNA is thought to silence target genes of the Notch pathway [47],[48]. However, downregulation of Notch signalling alone might not explain the synergism between *mir-7* and *Dl* overexpression in eye overgrowth as we did not detect reduction of the organizing signalling by Dl-Notch in these discs (Figure S3). Therefore, we sought to identify miR-7 target gene(s) that might be relevant to the cooperation with Dl-Notch signalling in eye overgrowth and tumourigenesis. As such, we systematically assayed a set of 39 *D. melanogaster* genes predicted to be miR-7 targets in silico (Table S1, [49]). We used RNA interference (RNAi) UAS-driven transgenes (UAS-IR) to downregulate candidate and previously validated miR-7 target genes *in vivo*. The *UAS-IR* transgenes silence specific mRNA transcripts by provoking their degradation, which is triggered by the generation of double-stranded RNA fragments complementary to the transcript driven by GAL4/UAS system [50],[51]. Here, we employed *ey-Gal4* to drive simultaneously the overexpression of the *UAS-IR* and the *UAS-Dl* transgene (Table S1).

We hypothesized that *mir-7* overexpression would be mimicked by endogenous downregulation of the functional relevant target gene(s) in the context of *Dl* overexpression. The assay would not, however, distinguish between a *bona fide* miR-7 target gene and those genes that are required normally for restricting tissue growth. To identify the former, we considered that a *bona fide* miR-7 target gene would not produce any effect when downregulated in the context of normal Notch signalling. Nevertheless, we took into consideration that RNAi silences mRNA more efficiently than microRNAs, and thus, we considered that UAS-IR lines of bona fide candidate genes would produce phenotypes similar to those of miR-7, or more severe. We tested candidate target genes predicted by several algorithms ([52]; see Materials and Methods) and that contain the conserved *Drosophila* miR-7 binding sites, which normally reduces the number of false positive target predictions.

**Figure 2:**
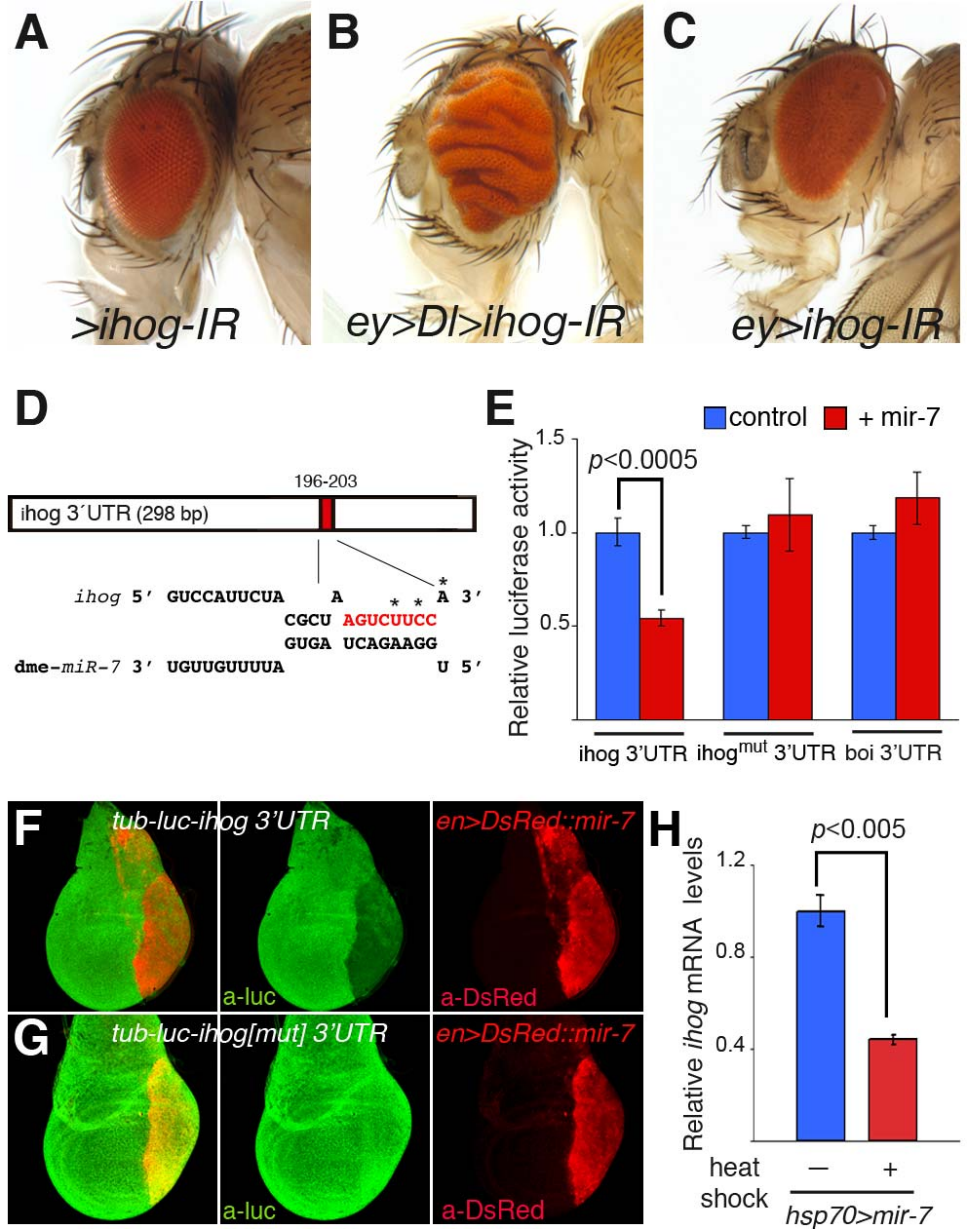
Tumourigenesis promoted by miR-7 via direct repression of *interference hedgehog* (*ihog*). (A–C) Adult heads of female control *UAS-ihog-IR* (A) and combinations of *UAS-ihog-IR* and *ey-Gal4* in the presence (B) or the absence (C) of the *Dl* transgene. (D) Computer predicted consequential pairing of *ihog* target region (top) and miRNA (bottom). The conserved seed match (8 mer) in the 3′UTR of *ihog* is in red. (E) Luciferase assay in *Drosophila* Schneider (S2) cells co-transfected with *mir-7* (red bars) or the empty vector (blue bars), together with a firefly luciferase vector containing the *ihog*3′UTR (*ihog*3′UTR), or the luciferase vector with mutations in the seed sequence (asterisks in *D*, *ihog^mut^3′UTR*) or control *boi*3′UTR (*boi*3′UTR). Firefly luciferase activity was measured 48 h after transfection and normalized against *Renilla* luciferase. The values represent the mean ± s.e.m. of three or four independent experiments. Differences in *ihog(mut)* and *boi* luciferase levels were not statistically significant between treatments. (F–G) Confocal images of mid third instar wing discs carrying the *tub-luc::ihog-3′UTR* (F) or the *tub-luc::ihog^mut^3′UTR* sensor (G) and overexpression of *mir-7* by *en-Gal4* (*en>DsRed::mir-*7, red) and stained with anti-luciferase antibody (green). (H) Differences in *ihog* mRNA levels assessed by RT-qPCR between *hsp70>mir-7* larvae subjected to heat shock treatment (red bar) or not (blue bar). Values represent the mean ± s.e.m. of three independent experiments. *P* values were calculated by the unpaired Student's *t* test.

Of the 39 candidate target genes assayed in conjunction with *Dl* overexpression, only reduction of two genes robustly cooperated with Dl-Notch signalling to provoke severely overgrown and folded eyes. A previously validated target of miR-7, *hairy* [48] was capable of converting *Dl*-induced mild overgrowth into tumour-like growth (Table S1). However, since miR-7 only very subtly reduces the expression of endogenous *hairy* and a GFP-3′UTR hairy sensor [48], we focused our interest on the gene, *interference hedgehog* (*ihog*), that when downregulated in *Dl*-overexpressing cells provoked robust overgrowth (Figure 2, Figure S4E–F,H, and Table S1).

Although not previously characterized as a target gene of miR-7, the downregulation of *ihog* by RNAi concomitant with the gain of *Dl* function consistently produced enlarged eye discs (Figure S4E–F) similar to that in eye discs co-expressing *Dl* and *mir-7* (Figure S3I–J), resulting in adults with overgrown and folded eyes (*ey>Dl>ihog-IR*: 80% of severe overgrown eyes, *n = *200; Figure 2B and Table S1). This phenotype was seen with the two independently generated *ihog-IR* transgenic lines available, both yielding identical results. Moreover, the expression of *ihog* RNAi alone during eye development did not alter the size or retinal patterning of this organ (*ey>ihog-IR*; Figure 2C). We confirmed that the *ihog-IR* transgenes inhibited *ihog* transcription 10-fold by quantitative reverse transcription-polymerase chain reaction (qRT-PCR; Figure S5A). Furthermore, the mRNA levels of *brother of ihog* (*boi*) were unaffected by these *ihog-IR* lines (Figure S5B). Thus, specific down-regulation of endogenous *ihog*, a predicted target of miR-7, facilitates overgrowth by *Dl* overexpression similar to those that develop when *mir-7* is overexpressed in this context (Figure 1, Figure 2, and Figure S4H).

### Validation of *Interference Hedgehog* as a Direct Target of miR-7 in Vitro and in Vivo

Since the *ihog* gene encodes a receptor of Hh in the embryo, including the imaginal eye disc [30], we assessed whether it is directly regulated by miR-7 in luciferase reporter-based cellular assays in vitro and in vivo (Figure 2). There is a single conserved miR-7 binding site in the 3′UTR of *ihog* (Figure 2D) and in *Drosophila* Schneider (S2) cells overexpressing *mir-7*, there was 45% less activity of a luciferase reporter containing the full-length *ihog* 3′ UTR downstream of the firefly luciferase coding region driven by the α-tubulin promoter (*tub-luc::ihog-3′UTR* Figure 2E and Figure S5C). By contrast, when the *ihog* 3′UTR construct carried point mutations in the miR-7 binding site (*tub-luc::ihog(mut)-3′UTR*), luciferase activity was the same as in control cells (Figure 2E). In addition, luciferase activity was unaffected by *mir-*7 overexpression in a control *tub-luc::boi-3′UTR* construct, indicative that the functional similar *boi* was not a target of miR-7 (Figure 2E).

In addition to the direct regulation of the *ihog* mRNA 3′UTR by miR-7 in vitro, there was specific in vivo repression of the *tub-luc::ihog-3′UTR* construct but not the *ihog* 3′UTR construct that carried the mutations in the seed sequence (Figure 2FG) and of an *ihog* 3′UTR eGFP sensor (*tub-eGFP::ihog-3′UTR*) but not a similar *boi* 3′UTR eGFP sensor (*tub-eGFP::boi-3′UTR*) (Figure S6AB) in the posterior compartment cells of third instar wing discs overexpressing *mir-7* driven by *engrailed* (*en*)*-Gal4*. Finally, we demonstrated that endogenous *ihog* mRNA was inhibited by miR-7 in vivo as heat shock induction of mature *mir-7* overexpression (*hsp70-Gal4 UAS-mir-7*) provoked a 55% reduction in *ihog* mRNA transcripts in larvae when assayed by qRT-PCR (Figure 2H and Figure S2D). Overall, these data provide convincing evidence that miR-7 is capable of directly repressing *ihog*, both in vitro and in vivo. Thus, the synergism between miR-7 and the Dl-Notch pathway activity in eye overgrowth would appear to be largely due to the silencing of *ihog*.

### *brother of ihog* Is Negatively Regulated by Notch Signalling during Eye Growth

**Figure 3:**
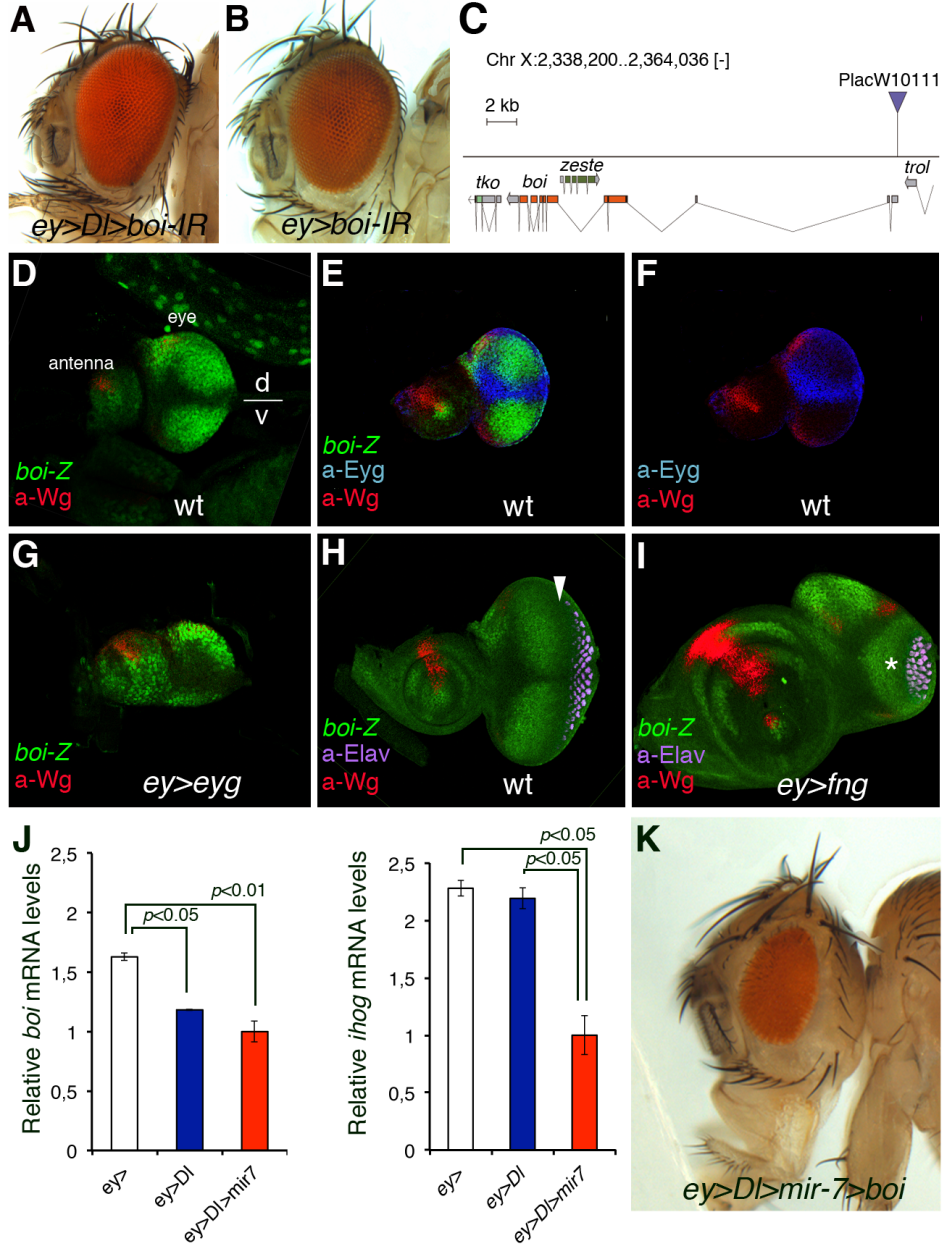
Notch signalling represses *brother of ihog* (*boi*) expression in the dorsal-ventral growth organizer in *Drosophila* eye. (A–B) Adult heads of female flies overexpressing *UAS-Dl* and/or *UAS-boi-IR* and *ey-Gal4*. (C) Map of *PlacW10111 P*-element insertion (triangle) into the *boi* locus. (D–I) *boi* expression in wild-type (D, E, F, and H) and Notch pathway mutant (G and I) eye-antennal discs. The patterning gene wingless (a-Wg, in red) serves to orient the eye disc in the dorsal (D)/ventral (V) axis. Expression of Boi (green) Hh co-receptor at the early third larval stage is repressed along the DV organizer (D and E), as defined by the expression of the DV organizer gene *eyg* (blue, E and F). Retinal differentiation (neuronal marker a-Elav, magenta) is first detected at the posterior end of the eye disc (to the right) and progresses in an anterior direction (H). The arrow points to the MF. (G and I) Expression of *boi-lacZ* (*boi-Z*, green) and wingless (a-Wg, red) in *ey>eyg* (G) and *ey>fng* (I) eye discs. The discs in (H) and (I) are from the same stage and magnification. The enlarged antennal disc in (I) is an effect of the undergrowth of the eye disc, caused in part by defective Notch activation in the D/V organizer due to *fng* overexpression. (J) qRT-PCR analyses of *boi* (left) and *ihog* (right) in *ey-Gal4* (white bar), *ey>Dl* (blue bar), and *ey>Dl>mir-7* (red bar) late third instar eye discs. Two independent experiments of three replicates are shown in each case. Data were normalized to *rp49*. mRNA isolated from 50 pairs of eye-antennal discs per genotype. Data analysed by a two-tailed unpaired *t* test. Error bars represent s.e.m. of three replicates. (K) Adult fly head showing no eye overgrown induced by *Dl* and *mir-7* when *boi* is overexpressed by a transgene (*UAS-boi*, 100% penetrance of rescue).

Although *boi* mRNA expression was not affected in the *ihog-IR* lines and Boi does not appear to be a target of miR-7, there is a well-documented functional overlap in the roles of Ihog and Boi. Moreover, genetic inactivation of both the *boi* and *ihog* genes is typically required to induce *hh* loss-of-function phenotypes [28],[30],[32]. However, unlike *ihog-IR*, we found that expressing an RNAi transgene against *boi* (*boi-IR* effectively reduces *boi* but not *ihog* mRNA levels by 65%; *p = *0.0005; Figure S5A–B) did not enhance *Dl*-induced eye overgrowth (*ey>Dl>boi-IR*; Figure 3A–B and Table S2). Since only the concomitant loss of both *ihog* and *boi* leads to a loss of eye tissue [30], we reasoned that a similar situation might occur with respect to the *ihog-IR*-induced severe eye overgrowth (Figure 2B). Consequently, we verified the status of *boi* transcription in relation to eye disc growth. Interestingly, the spatial domain of *boi* in the developing eye disc in vivo using a ß-galactosidase enhancer trap inserted in *boi* (*P-*lacW stock *10111*; Figure 3C) unveiled that *boi* is expressed nonuniformly in the region anterior to the MF with a weakest expression within the DV organizer (Figure 3D–E,H: the MF is denoted by an arrow in H). Indeed, in eye discs double labelled with anti-Eyg (a DV organizer-specific response gene and an obligatory Notch's effector in eye growth [13],[53]) and anti-ß-galactosidase (*boi-lacZ* in green), we found that the expression of Eyg precisely borders the “negative” domain of *boi* (Figure 3E–F). This led us to speculate that expression of *boi* is negatively regulated by Notch-Eyg at the growth-promoting organizer, which we investigated by monitoring the spatial domain of *boi-lacZ* in mutants of the DV organizer and by assessing *boi* mRNA levels by qRT-PCR analyses.

We assayed the ubiquitous expression of the Notch DV organizer transcriptional effector Eyg, which provokes a wider DV organizer domain [13],[53] and observed an extended domain lacking *boi-lacZ* expression under these conditions (Figure 3G). Conversely, the ubiquitous expression of the modulator *fringe* (*fng*) causes defective Notch receptor activation by its ligands and results in the thinning or loss of the DV organizer [53]–[55]. Under these conditions, the expression of *boi* was uniform throughout the eye disc due to the absence of the “central domain” that represses this gene in wild-type eye discs (Figure 3I). Thus, *boi* is negatively regulated by Notch's organizer activity or it at least reflects this activity negatively. Since Eyg encodes a transcriptional repressor [14],[15], it may directly repress *boi* transcription. This Hh co-receptor does contain a consensus Eyg-binding site for repression (TCACTGA [14]) at position chrX: 2.359.784, although we could not validate the direct binding of Eyg to the *boi* promoter region by chromatin immunoprecipitation (unpublished data). Nevertheless, it is possible that Eyg might bind through other nonconsensus sites. Furthermore, qRT-PCR analyses confirmed downregulated *boi* but not *ihog* transcripts in eye discs overexpressing *Dl* transgene alone by *ey-Gal4* (ey*>Dl*; left in Figure 3J). Importantly, both *boi* and *ihog* mRNA levels were downregulated in eye discs that co-expressed *Dl* with the microRNA *mir-*7 (*ey>Dl>mir-7*; Figure 3J). *boi* and *ihog* RNA was isolated from whole eye-antennal disc complexes; thus, the mRNA levels are the sum of all regions of the discs, including the antenna, which is not affected by *ey>*Dl or *ey>mir-*7. Hence expression differences with control may be significant underestimations of the actual differences of each gene in the eye disc parts in the different genotypes. Nevertheless, the qRT-PCR comparisons between the different genotypes showed a trend in *boi* and *ihog* expression response to Dl overexpression that explains the cooperation between the miR-7 and *Dl* signalling, since there is the concomitant downregulation of the two functionally redundant Hh receptor genes, *ihog* and *boi*.

Animals homozygous for mutations in *ihog* and *boi* exhibit a phenotype typical of the loss of *hh* function (e.g., [30]). The defect in *ihog^−^ boi^−^* animals can be rescued by expressing a *UAS-ihog::myc* transgene with weak constitutive expression in the absence of Gal4 activity [30]. Surprisingly, we could not overcome overgrowth by *mir-7/Dl* using this transgene (unpublished data). This may perhaps reflect that the elevated levels of Ihog expected by Gal4-induced expression of the transgene may exert a dominant negative effect on Hh signalling [31]. A *boi* transgene (*UAS-boi*) [56] fully suppressed the overgrowth induced by the combination of *mir-7/Dl* (Figure 3K, 100% penetrance, *n = *100). The same result was obtained using the *EP(X)1447(boi)* that misexpresses endogenous *boi* gene (unpublished data).

### Blocking Core Hedgehog Signalling Components or Expressing Ci/GLI Repressor Mimics the Effect of the MicroRNA in Delta-Induced Tumourigenesis

To confirm that silencing Hh signal transduction facilitates a tumorigenic response to Dl-Notch overactivation, we next assayed the effects of directly downregulating core Hh signalling elements with RNAi transgenes driven by *ey-Gal4*, including *smo*, *ci*, and *hh* itself. As noted above, Gal4 drives expression throughout early eye disc development anterior to the MF, a region of undifferentiated proliferating eye cells that act on signals from the Notch-mediated DV organizer, and Gal4 expression terminates before cells exit the cell cycle at the MF [54]. We down-regulated each of these Hh signalling components by RNAi, assaying several independent lines in which the use of *ey-Gal4* avoided the possible effects of a loss of Hedgehog signal transduction on retinal differentiation that might confound the results (Table S2).

**Figure 4:**
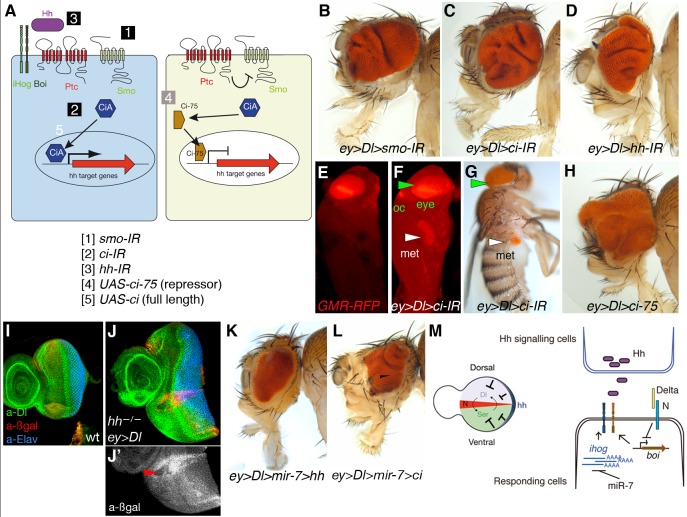
Downregulation of elements in the Hh pathway or overexpression of the repressor form of *ci* co-operates with *Dl* overexpression to trigger tumour growth in the *Drosophila* eye. (A) Schematic representation of Hh signalling and the *UAS* transgenes used to downregulate by RNAi (IR) or activate Hh pathway components. (B–D, H, and K–L) Representative adult heads of female flies of combinations of the indicated *UAS* transgenes and *ey-Gal4* are shown. (E–F) Fluorescent images of *Drosophila* pupae of sibling control (*ey>Dl*, E) or *ey>Dl>ci-IR* (F). (G) Adult fly of *ey>Dl>ci-IR* with a metastatic (met) growth in the abdomen. Eye tissue in the endogenous site (green arrowheads) and distant site (white arrowheads) is labelled by the retinal-specific GMR-myrRFP marker (E, F) or the retinal-specific red pigments (G). (I–J′) Third instar wild type of sized eye disc (I) and *ey>Dl* eye disc carrying clones of *hh^AC^* labelled by the absence of *arm-lacZ* (ßgal, red in J and grey in J′). Arrowhead points to a clone and its associated twin spot (high red staining). (M) Model of antagonistic interaction between Hh and Notch signalling in normal eye imaginal disc (left) and model of regulatory interactions among the microRNA, Notch pathway, and the Hh receptors *ihog* and *boi* (right). Genotype in (J) is: *yw ey-Flp; ey-Gal4 UAS-Dl/+; FRT82B hh^AC^/FRT82B arm-lacZ*.

The downregulation of *smo* (80% flies exhibited eye tumour-like growth, *n* >200), *ci* (100%, *n*>200), or *hh* (30%–100%, *n*>200) in conjunction with *Dl* overexpression provoked a tumour phenotype similar to that of RNAi of *ihog* but stronger than the overexpression of *mir-7* (compare Figure 1 and Figure 2 with Figure 4B–D; see also Table S2). Furthermore, downregulation of *ci* by RNAi (*ci-IR*) by *ey-Gal4* stimulated a metastatic overproliferation of eye tissue in the context of the *Dl* gain of function, resulting in flies with secondary eye growths within the thorax and abdomen (Figure 4F–G and Table S2). This invasive overgrowth is also observed when *Dl* and the *ci* RNAi transgene are expressed in the wing imaginal discs by the *dpp-*Gal4 (Figure S7). Like the *mir-7* and *ihog-IR* lines (Figure 1L and Figure 2C), none of the above RNAi lines were capable of inducing overgrowth by themselves.

In all contexts, in the absence of Hh signal or its reception, the transcription factors of the Ci/Gli family (in *Drosophila*, full-length Ci-155) can be proteolytically processed into a truncated (N-terminal 75 kDa in *Drosophila*—Ci-75) transcriptional repressor of the Hh pathway (Ci, Gli3, and to a lesser extent Gli2) (Figure 4A). The bifunctional nature of Ci [57]–[59], and of the mammalian homologues Gli2 and Gli3, could fulfil oncogenic or tumour suppressor roles in function of the status of the Hh signalling. As *ci-IR* downregulates both activator and repressor forms, we next assessed the contribution of the truncated Ci repressor that forms in the absence of Hh signalling, testing the effect of overexpressing *Dl* with a transgene of the constitutive Ci repressor form (*UAS-ci-75*). Co-overexpression of *Dl* and *ci-75* induced eye tumour-like growth in 75% of fly eyes (*ey>Dl>ci75*; *n* = 100; Figure 4H), in contrast to the overexpression of Ci full length (*UAS-ci*) that acts as an activator in Hh receiving cells and did not provoke eye tumour (unpublished data).

To further verify these findings with the RNAi transgenes, we generated marked clones of cells homozygous for *hh^AC^* (a null allele) in the *ey>Dl* background (*hh^AC^/hh^AC^ ey>Dl*; Figure 4J). Eye discs carrying small patches of *hh^AC^* cells were 170% larger than control wild-type eye discs (Figure 4I) and 126% larger than *ey>*Dl without *hh^AC^* clones eye discs (see Figure S4B). Using the MARCM technique [60], we also examined GFP-labelled clones of cells overexpressing *Dl* and homozygous for *smo^3^* (an amorphic allele) (*smo^3^/smo^3^tub>Dl>GFP*; Figure S8). Whereas clones of *smo^3^* do not delay the MF [61] and clones of *Dl-*expressing cells normally cause autonomous advancement of the MF [62], we found that clones of *smo^3^ Dl-*expressing cells led to advancement of the MF also in surrounding wild-type cells (Figure S8B) and the disc was overall overgrown (unpublished data). The advanced MF is seen in *ey>Dl* eye discs with downregulation of Hh signalling via overexpression of *mir-7* or direct downregulation via RNAi transgenes (Figures S3 and S4). Thus, interfering with Hh signalling exacerbates the organizing activity of Dl-Notch signalling in eye imaginal discs and can foster invasive tumour growth (Figure 4F–G, Figure S7C–D, and Table S2).

### Increasing Hedgehog Signal Prevents Tumourigenesis by Delta and miR-7

In normal early eye development, when the Notch organizer induces a dramatic increase in cell proliferation in the disc, *hh* gene is expressed in a thin line of cells along the eye disc margin ([19],[20],[25]; see Figure S1). Previously, it has been shown that clones of eye disc cells lacking PKA, Ptc, or Cos2 proteins that normally prevent the inappropriate activation of Hh signal transduction exhibit within the clone a growth-disadvantage and are eliminated by apoptosis [26]. This negative influence of Hh signal was also hinted at by the small eye defect associated with overexpression of *UAS-hh* by *ey-Gal4* [25] and is complementary to our findings.

The Ihog/Boc family proteins normally enhance Hh binding to Ptc, the 12-pass transmembrane protein involved in sensing extracellular Hh concentrations. Binding of Hh to Ptc relieves inhibition of Smo by Ptc and blocks the production of Ci repressor. Hence, the downregulation of *ihog/boi* levels by Dl/miR-7 (see Figure 3J) might reduce the interactions of Hh with Ptc. We therefore investigated whether increasing Hh signal via a *UAS-hh* transgene to counterbalance *ihog/boi* deficit could rescue the overgrowth by *Dl*/*mir-7*. Indeed, we detected significant reduction in eye size in flies *ey>Dl>mir-7>hh* (Figure 4K; 100% rescue, *n*>100; see Figure S9 for scheme of genetic test for rescuing experiment) and also in flies that expressed Ci full length (*ey>Dl>mir-7>ci*; Figure 4L). Note that when Ci full length is expressed in the context of *Dl* and *mir-7* overexpression, although many eyes are substantially reduced in size they still exhibit abnormal patterned growth (see Figure 4L) and other exhibited enhanced tumorigenesis. We interpret these findings as Ci full length can be converted into the repressor form owing to the reduced Hh signalling caused by Dl and miR-7 depletion of *ihog* and *boi*.

Hh signal stimulates the maturation of Ci full length into a short-lived nuclear activator, while the PKA negative regulator opposes this event and when mutated results in constitutive Hh pathway activity. The undergrowth defect of knock-down of *pka* by RNAi expression in the *Dl* overexpressing eye discs (*ey>Dl>pka-IR*; Table S2) further support the tumour suppressor activity of Hh pathway in the context of gain of Dl-Notch signalling in the context of the eye primordium. We suggest here that in healthy flies the release of Hh by these eye disc marginal cells sets eye size in conjunction with the Dl-Notch organizer (Figure 4M, left scheme), and thereby dampening Hh signalling in the context of Dl overexpression (Figure 4M, right) fosters the developing eye tumours or overgrowth beyond the normal eye size.

### Hedgehog Signal Transduction Also Attenuates Delta Signalling and Overgrowth in the Wing

**Figure 5:**
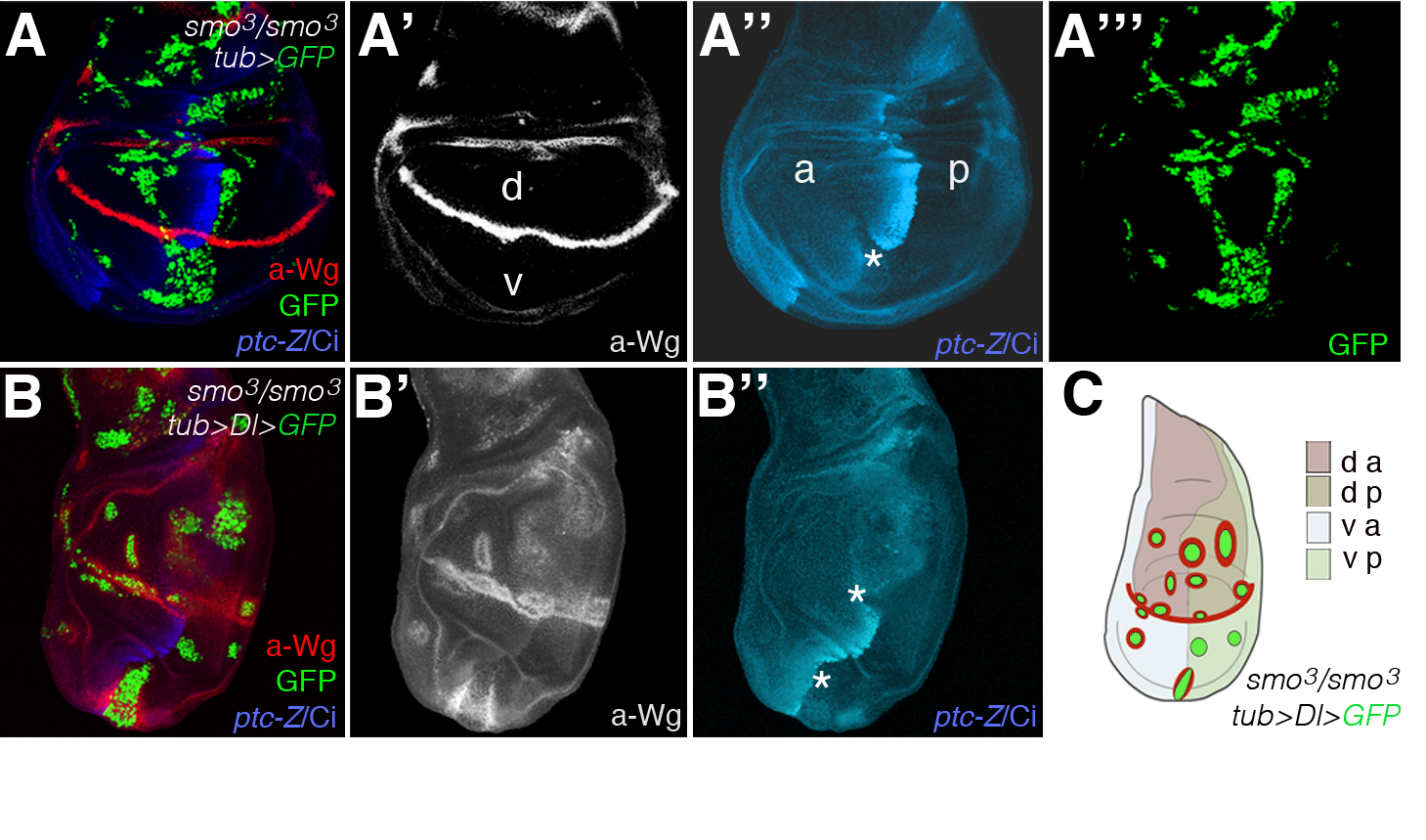
Failure to transduce the Hh signal due to mutations in smoothened enhances Dl-Notch signalling activity in the wing. (A–B″) Confocal images of wing discs bearing MARCM GFP (green)-labelled clones homozygous for *smo^3^* without (A) or with (B) *Dl* overexpression. Single channel images are also shown. Mosaic discs were stained for Wg (red in A and B, and grey in A′ and B′), and Ci (blue) and Ptc-lacZ (Ptc-Z, blue). (C) A schematic summary of clones in (B). Asterisks in (A″) and (B″) point to “posteriorly” situated clones that were of anterior origin as denoted by the failure to induce Ptc and the low levels of Ci protein (white line delineates the AP boundary in the discs in B). Clones were generated at 24–42 h after egg laying (AEL) by a 1 h heat shock at 37°C (*n = *60 clones analysed). Genotypes: (A) *yw hsp70-Flp tub-G4 UAS-GFP; tub-Gal80 FRT40A/smo^3^ FRT40A ptc-lacZ* and (B) *yw hsp70-Flp Tub-G4 UAS-GFP; Tub-Gal80 FRT40A/smo^3^ FRT40A ptc-lacZ; UAS-Dl/+*.

Wing disc growth and patterning is also organized by Hh and Notch-mediated organizers [2], with Hh secreted by cells in the posterior (P) compartment inducing short-range targets in anterior (A) cells near the AP boundary (e.g., *ptc*, blue staining in Figure 5A) [63],[64]. Notch signalling is activated locally along the DV boundary by its ligands Dl and Serrate (Ser), and it induces symmetric expression of targets in boundary cells (e.g., *wg*, red staining in Figure 5A; reviewed in [2]). Hence, we investigated whether the antagonistic interaction between loss of Hh and gain of Notch apparent in the eye imaginal discs can also be applied to the wing discs.

*Dl-*expressing clones in the wing induce ectopic *wg* expression in D cells, where the *fringe* gene is expressed, whereas ventrally situated clones did not activate *wg* (e.g., [65]–[69]). Enhancing Dl activity by co-expressing *Dl* with the E3 ubiquitin ligase *Neuralized*, which promotes the endocytosis and signalling activity of Dl, can induce *wg* in ventrally situated clones [69]. Hence, we assayed ectopic induction of *wg* to examine *Dl* activity in *smo^3^/smo^3^* clones. As shown in Figure 5, we found that ventrally situated A cells homozygous for *smo^3^* and expressing *Dl* expressed high levels of Wg, similar to the levels of Wg induced by dorsally situated clones, in contrast with most *smo^3^ Dl-*expressing clones situated ventrally in P cells away from the boundary (Figure 5B–C) or clones of *smo^3^* cells that do not overexpressed *Dl* (Figure 5A). Nonautonomous overgrowth is also evident in ventrally situated clones of *smo^3^/smo^3^ Dl-*expressing (Figure S8C). Clones of *smo^3^* cells abutting the AP boundary often sort to the P compartment territory [70],[71]. MARCM clones do not label the twin spot (*smo^+^/smo* ^+^); therefore, the inference that the clones at the AP boundary (asterisks in Figure 5A″–B′) are of anterior origin is supported by the finding that they retain anterior features (low levels of Ci protein). Loss of *smo* activity in A cells at the boundary fail to up-regulate Ci expression and do not induce *ptc* transcription. These clones cause an anterior shift in the distribution of *ptc* and up-regulated Ci non-cell-autonomously [64]. We occasionally found ambiguously positioned clones of *smo^3^/smo^3^ tub>Dl* cells in which the anterior part of the clone exhibited ectopic *wg* expression while the posterior of the clone did not (Figure S8D). Taken together, these findings show that *Dl-*expressing cells unable to transduce the Hh signal behave as they express hyperactivated Dl. Coupled with the analysis of RNAi transgenes, these results confirm that the loss of Hh signalling enhances Dl-Notch signalling activity.

### Loss of Hedgehog Signalling in miR-7 Overexpression in the Wing

**Figure 6:**
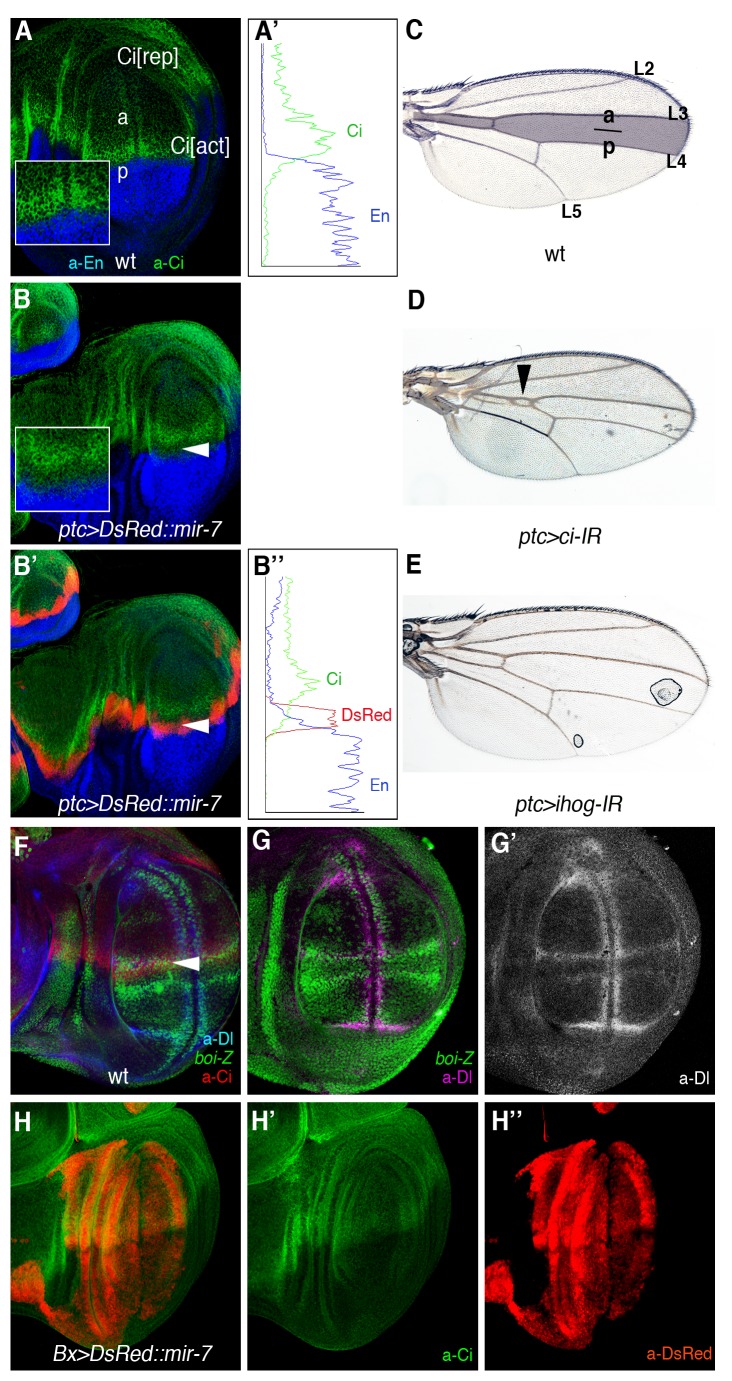
miR-7 silencing of Hh signalling explains the L3–L4 fusion defects in the wing. (A) Ci protein (green) is distributed across the entire anterior (A) compartment of the discs. Hh signals from posterior (P) cells induce high levels of Ci in cells along the AP border, and they block Ci proteolysis into the repressor form (Ci[rep]), thereby allowing the Ci activator (C[act]) to accumulate. (B–B′) Overexpression of *mir-7* denoted by red labelling (*UAS-DsRed::mir-7*) driven by *patched (ptc)-Gal4* downregulates Hh signalling as visualized by low Ci levels (green; white arrowhead). Insets show magnifications. Engrailed (En) staining in blue serves to mark the P compartment in (A–B″). Plots of fluorescence intensity profiles of the anterior-posterior compartments from the WT (A) and *ptc>DsRed::mir-7* (B′) discs are shown in (A′) and (B″), respectively. Green trace, Ci; blue trace, En; red trace, DsRed. (C) Adult wild-type wing. The shaded area denotes the domain of expression of the *ptc-Gal4* reporter. (D) *ci-IR* expression by *ptc-Gal4* mimicking the L3–L4 fusion defect seen in adult wings that is caused by *mir-7* overexpression (compare with Figure 1G). (E) Adult wing expressing *ihog-IR* driven by *ptc-Gal4*. (F–F″) The expression of *boi-lacZ* (green) defines all longitudinal veins (L2–L5). Note the high *boi-*lacZ (green in F) expression along L3, marked by high Ci (red in F) and Dl (magenta in F″). (G–G″) Overexpression of *mir-7* (in red) by *Bx-Gal4* did not alter Ci protein levels (green, white arrowhead).

microRNAs are thought to regulate multiple target genes; however, often when tested in vivo, it is a subset or a given target that function as the major effector of the activity of the microRNAs in a given cellular context. We asked whether our identification of *ihog* as a key target of miR-7 during Dl-mediated tumorigenesis in the eye might reflect endogenous roles of the microRNA in other tissues. Previously, misexpression of *mir-7* driven by *ptc-Gal4* (*ptc>mir-7*) produces wing margin notches, and a reduction of the space between vein L3 and L4 ([48]; see [72]). Both of these phenotypes have been attributed to defects in Notch signalling [48],[73], although we noted that L3–L4 fusion is very reminiscent to the phenotype produced by *hh* loss-of-function mutations, including that associated with the ci^Cell^ mutation that produces a truncated form of Ci, which behaves as a constitutive repressor [59]. Indeed, we observed a clear downregulation of Ci protein levels in cells in *ptc>mir-7* (Figure 6A–B″), which are precisely the cells receiving endogenous Hh signals and that upon normal Hh reception stabilize Ci protein levels and prevent the conversion of Ci-155 into truncated Ci repressor. Plots of fluorescence intensity profiles from the wild-type and *ptc>mir-7* discs are shown in Figure 6A′ and B″. The weak downregulation of Ci by mild RNAi expression using *ptc-Gal4* mimicked the L3–L4 fusion defect of *ptc>mir7* (Figure 6C–D). Depleting *ihog* by RNAi driven by *ptc-Gal4* did not produce a defect as *mir-7* overexpression (Figure 6E). The lack of effect of *ihog* RNAi is almost certainly due to the activity of the other Hh co-receptor, *boi*, which is expressed at high levels in the wing margin and in the presumptive L3 vein territory (*boi-lacZ* in green; Figure 6F). These results raised the possibility that like *ihog*, *ci* is also a direct target of miR-7. Indeed, c*i* mRNA does contain a presumptive miR-7 binding site in the *ci* 3′UTR, although this site is not conserved across *Drosophila* species. Thus, the Ci low protein levels in *ptc>mir-*7 wing discs could reflect the direct repression of *ci* by the microRNA or the dampening of Hh signalling response by the miR-7-mediated downregulation of *ihog* or both. More consistently with indirect regulation of Ci by miR-7, we observed no change in Ci protein levels in wing discs ectopically expressing the *mir-7* away from the normal Hh secreting cells (the P compartment cells marked by the absence of Ci (green) in Figure 6G). In this experiment, we used the *Beadex* (*Bx*)*-Gal4* driver, with the Bx domain labelled by DsRed because of the *UAS-DsRed::mir-*7 transgene (Figure 6G). Therefore, either Ci is not a target of miR-7 or this regulation is context dependent. It is generally considered that when an individual miRNA affects the expression of various proteins in the same pathway, it does so in a rather mild manner [74]. Thus, the relevance of co-regulation of *ihog* and *ci* by miR-7 in Hh receiving cells deserves further analysis given that the human counterparts of these genes (CDO, BOC, and Gli3) also contain binding sites for human miR-7.

## Discussion

A challenge to understand oncogenesis produced by pleiotropic signalling pathways, such as Notch, Hh, and Wnts, is to unveil the complex cross-talk, cooperation, and antagonism of these signalling pathways in the appropriate contexts. Studies in flies, mice, and in human cell cultures have provided critical insights into the contribution of Notch to tumourigenesis. These studies highlighted that Notch when acting as an oncogene needs additional mutations or genes to initiate tumourigenesis and for tumour progression, identifying several determinants for such co-operation (e.g., [7],[8],[10],[24],[44],[75]–[79]). The identification of these co-operative events has often been knowledge-driven, although unbiased genetic screens also identified known unanticipated tumour-suppressor functions. In this sense, we describe here a conserved microRNA that cooperates with Notch-induced overproliferation and tumour-like overgrowth in the *D. melanogaster* eye, miR-7. Alterations in microRNAs have been implicated in the initiation or progression of human cancers (e.g., [80]–[84]), although such roles of microRNAs have rarely been demonstrated in vivo (e.g., [85]–[88]). In addition, by identifying and validating functionally relevant targets of miR-7 in tumourigenesis, we also exposed a hitherto unsuspected tumour suppressor role for the Hh signalling pathway in the context of the oncogenic Notch pathway. Given the conservation of the Notch and Hh pathways, and the recurrent alteration of microRNAs in human cancers, we speculate that the genetic configuration of miR-7, Notch, and Hh is likely to participate in the development of certain human tumours.

In human cancer cells, miR-7 has been postulated to have an oncogene [89],[90] or a tumour suppressor functions [91]–[96] that may reflect the participation of the microRNA in distinct pathways, due to the regulation of discrete target genes in different cell types, such as *Fos* [97] in mouse, and *Pak1* [91], *IRS-2* [92], *EGFR* [92],[93], *Raf-1* [93], α*-synuclein* [98], *CD98* [99], *IGFR1* [94], *bcl-2* [100], PI3K/AKT [101],[102], and *YY1* [103] in humans.

In *Drosophila*, multiple, cell-specific, targets for miR-7 have been previously validated via luciferase or in vivo eGFP-reporter sensors or less extensively via functional studies [47],[49],[73],[104]–[107]. Although microRNAs are thought to regulate multiple target genes, when tested in vivo it is a subset or a given target that predominates in a given cellular context. Indeed, of the 39 predicted miR-7 target genes tested by direct RNAi, only downregulating *ihog* with several RNAi transgenes (*UAS-ihog-IR*) fully mimicked the effect of miR-7 overexpression in the transformation of *Dl*-induced mild overgrowth into severe overgrowth and even tumour-like growth. Moreover, we confirmed that endogenous *ihog* is directly silenced by miR-7 and that this silencing involves direct binding of the microRNA to sequences in the 3′UTR of *ihog* both in vivo and in vitro.

Nevertheless, other miR-7 target genes may contribute to the cooperation with Dl-Notch pathway along with *ihog*, such as *hairy* and *Tom*. While miR-7 can directly silence *hairy* in the wing, this effect has been shown to be very modest [48], and thus, we consider that while *hairy* may contribute to such effects, it is unlikely to be instrumental in this tumour model. Indeed, the loss of *hairy* is inconsequential in eye development [108], although retinal differentiation is accelerated by genetic mosaicism of loss of *hairy* and *extramacrochaetae* [108]. *hairy* is a target of Hh [18],[21] that negatively sets the pace of MF progression. It is unclear how Hairy might contribute to Dl-induced tumourigenesis.

The RNAi against *Tom* produced overgrowth with the gain of *Dl* albeit inconsistently and with weak penetrance, where one RNAi line did not modify the Dl-induced overgrowth and the other RNAi line caused tumours in less than 40% of the progeny (Table S1). Tom is required to counteract the activity of the ubiquitin ligase Neuralized in regulating the Notch extracellular domain, and Dl in the signal emitting cells. These interactions are normally required to activate Notch signalling in the receiving cells through lateral inhibition and cell fate allocation [109]. However, although it remains to be shown whether similar interactions are active during cell proliferation and growth, the moderate enhancement of *Dl* that is induced when *Tom* is downregulated by RNAi suggests that miR-7-mediated repression of *Tom* may contribute to the oncogenic effects of miR-7 in the context of *Dl* gain of function, along with other targets such as *ihog*.

Conversely, while the target genes of the Notch pathway, *E(spl)m3* and *E(spl)m4* [48] as well as *E(spl)mγ*, *Bob*, *E(spl)m5*, and *E(spl)mδ* [60], have been identified as direct targets of miR-7 in the normal wing disc via analysis of 3′UTR sensors, there was no evidence that *HLHm3*, *HLHm4*, *HLHm5*, *Bob*, and *HLHmγ* are biological relevant targets of miR-7 in the Dl overexpression context. *HLHmδ* RNAi produced inconsistent phenotypes in the two RNAi transgenic lines available, causing tumour-like growth at very low frequency in only one of the lines (Table S1). We also did not obtain evidence that miR-7 provoked overgrowth by targeting the ETS transcription factor in the EGFR pathway AOP/Yan (Table S1), a functionally validated target of the microRNA miR-7 during retinal differentiation [47]. Neither had we obtained evidence that RNAi of *atonal* provoked eye tumours with Dl overexpression (Table S1), although a strong inhibition via expression of a fusion protein Atonal::EN that converts Atonal into a transcriptional repressor has been shown to be sufficient to trigger tumorigenesis together with Dl [24]. Thus, we reasoned that given that microRNA influenced target genes only subtly (even when using ectopic expression), it is possible that downregulation of *atonal* contributes to the phenotype along with the other targets.

In conclusion, we have identified cooperation between the microRNA miR-7 and Notch in the *D. melanogaster* eye and identified and validated *ihog* as a direct target of the miR-7 in this context and have identified *boi* as a target of Notch-mediated activity at the DV eye organizer, although it remains whether this regulation is direct or indirect. We also uncovered a hitherto unanticipated tumour suppressor activity of the endogenous Hh signalling pathway in the context of gain of Dl-Notch signalling (Figure 4) that is also apparent during wing development (Figure 5).

Hh tumour suppressor role is revealed when components of the Hh pathway were lost in conjunction with a gain of Dl expression in both the eye (Figure 4) and wing (Figure 5 and Figure S8) discs. Hh and Notch establish signalling centres along the AP and DV axes, respectively, of the disc to organize global growth and patterning. Where the organizer domains meet, the Hh and Notch conjoined activities specify the position of the MF in the eye disc and the proximodistal patterning in the wing disc [25],[47],[48]. We unveil here that in addition antagonistic interaction between the Hh and Notch signalling might help to ensure correct disc growth. Thus, we show that Hh signalling limits the organizing activity of Dl-Notch signalling (Figure 4, Figure 5, and Figure S8). Although it is often confounded whether Dl-Notch signalling instructs overgrowth by autonomous or nonautonomous (i.e., DV organizers) mechanisms, our findings uncover that loss of Hh signalling enhances a noncell autonomous oncogenic role of Dl-Notch pathway (Figure 4J and Figure S8D).

To date, Hh has not yet to be perceived as a tumour suppressor, although it is noteworthy that human homologs of ihog, CDO, and BOC were initially identified as tumour suppressors [42]. Importantly, both CDO and BOC are downregulated by RAS oncogenes in transformed cells [42] and their overexpression can inhibit tumour cell growth in vitro [42],[110],[111]. Since human RAS regulates tumourigenesis in the lung by overexpressing miR-7 in an ERK-dependent manner [90], it is possible that RAS represses CDO and BOC via this microRNA. Indeed, the 3′UTR of both *CDO* and *BOC* like *Drosophila ihog* contains predicted binding sites for miR-7 (www.targetscan.org). There is additional clinical and experimental evidence connecting elements of the Hedgehog pathway with tumour-suppression. The function of *Growth arrest specific gene 1* (*GAS1*), a Hh ligand-binding factor, overlaps that of *CDO* and *BOC* [39],[41] and its downregulation is positively associated with cancer cells [94] and melanoma metastasis [112], while its overexpression inhibits tumour growth [113]. More speculative is the association of some cancer cells with the absence of cilium, a structure absolutely required for Hh signal transduction in vertebrate cells [27].

Given the pleiotropic nature of Notch, Wnts, BMP/TGFß, Ras, and Hh signalling pathways in normal development in vivo, we speculate that competitive interplay as that described here between Notch and Hh may not be uncommon among core growth control and cancer pathways that act within the same cells at the same or different time to exert multiple outputs (such as growth and cell differentiation). Moreover, context-dependent tumour suppressor roles could explain the recurrent, unexplained, identification of somatic mutations in Hh pathway in human cancer samples (e.g., [43]). Indeed, our findings stimulate a re-evaluation of the signalling pathways previously considered to be exclusively oncogenic, such as the Hh pathway.

## Materials and Methods

### Drosophila Husbandry

The *GS(2)518ND2* line was isolated in a genetic screen for enhancers or suppressors of a mild overgrown eye phenotype induced by *Dl* overexpression when driven by the eye-specific *ey-Gal4* driver (*ey-Gal4 UAS-Dl*). The *PlacWP1O111* stock was a generous gift from Dr. C. Klambt (Munster University, Munster, Germany), and the other *Drosophila* stocks used here were: *UAS-mir-7* and *UAS-DsRed::mir-7* [47], *UAS-boi* [56], *UAS-ci* [57], and *UAS-ci-75* [58],[59]. A detailed description of the stocks and transgenic flies used in this study can be found at http://flybase.org/ for *ey-Gal4*, *ptc-Gal4*, *en-Gal4*, *hsp70-Gal4*, *Bx-Gal4*, *UAS-Dl*, *UAS-fng*, *UAS-hh*, *UAS-eyg*, *EP(X)1447 (boi)*, *hh^AC^*, and *smo^3^*or at http://flystocks.bio.indiana.edu/ and http://stockcenter.vdrc.at/control/main for the BDSC and VDRC RNAi stocks, respectively. Clones of *hh^AC^* surrounded by *Dl-*expressing tissue (Figure 4J) were generated by the ey-Flp in eye-antennal imaginal discs of the genotype: *yw ey-Flp; ey-Gal4 UAS-Dl/+; FRT82B hh^AC^/FRT82B arm-lacZ*. In Figure S8, the MARCM GFP-labelled clones of *smo^3^/smo^3^* only or *smo^3^/smo^3^tub-Gal4 UAS-Dl* cells were induced by 1 h heat shock at 37°C at 48–72 h AEL in larvae: *y w tub-Gal4 UAS-GFP hsp70-FLP122; smo^3^ FRT40A ptc-lacZ/tub-Gal80 FRT40A* and *y w tub-Gal4 UAS-GFP hsp70-FLP122; smo^3^ FRT40A ptc-lacZ/tub-Gal80 FRT40A; UAS-Dl/*+, respectively.

All the combinations of *Gal4*, *GS*, and the different *UAS* transgenic lines and mutants were raised at 26.5°C.

### GS-Element and PlacW Mapping

Genomic DNA flanking the *P*-element insertion in the *GS(2)518ND2* and the *PlacWP1O111*stock were recovered by inverse PCR using the Pwht1/Plac1 and Plw3-1/Pry 4 primers, respectively (http://www.fruitfly.org/about/methods/inverse.pcr.html), and they were subsequently sequenced. A BLAST search with the sequence produced perfect matches to the genomic region on chr2R:16491078 for *GS(2)518ND2* and on chrX: 2364036 for *PlacWP1O111*.

### Quantitative Reverse Transcriptase PCR (qRT-PCR)

To assess the levels of *ihog* or *boi* mRNA when the *mir-7* or RNAi lines were activated by Gal4, we performed qRT-PCR experiments using RNA isolated from wandering third instar larvae of the *hsp70-Gal4* genotype crossed with transgenic lines (*UAS-mir-7*, *UAS-ihog-IR*, or *UAS-boi-IR*) directly or following heat shock (an hour at 37°C followed by 6 h at 25°C). Total RNA from 50 pairs of eye-antennal discs was extracted for experiments in Figure 3J. All tissue samples were stored in RNAlaterTissueProtect Tubes (Qiagen) until used and mature *mir-7*, *ihog*, or *boi* mRNA levels were assessed by qRT-PCR. Note that RNA was isolated from whole eye-antennal disc complexes; thus, the levels of *boi* and *ihog* mRNA expression are the sum of all regions of the discs, including the antennal disc part that might not be unaffected by the expression of *ey-Gal4*. Thus, expression differences between the control and *Dl* and/or *mir-7* overexpressing eye-antennal disc complexes may be significant underestimations of the actual differences in the relevant eye disc part in each genotype. To analyse mature *mir-7* expression, we used *mir-7*-specific primers from the TaqMan MicroRNA Assays (Applied Biosystems), together with the TaqMan MicroRNA Reverse Transcription Kit (Applied Biosystems) and TaqMan Universal PCR Master Mix (Applied Biosystems). The *mir-7* levels were normalized to *U14* snRNA. To determine *ihog* and *boi* mRNA levels, we used SuperScript First-Strand Synthesis System for RT–PCR (Invitrogen) and SYBR Green PCR Master kit (Applied Biosystems), according to the manufacturer's instructions. The cDNAs were amplified using specific primers designed using the ProbeFinder software by Roche Applied Science, and *rp49* was used as a house-keeping gene for normalization.

Primer sequences used in this study include the following: *ihog*, forward primer 5′-TCAGTCTAAAATCCCATAATAAGTGC-3, reverse primer 5′-AAACCGGAATTGCTTCGAG-3′; *boi*, forward primer 5′-TGCCTAAAGAGACGGGAAAA-3′, reverse primer 5′-ATGTGTTCCAATTGCGGTTT-3′; and *rp49*, forward primer 5′-TGTCCTTCCAGCTTCAAGATGACCATC-3′, reverse primer 5′-CTTGGGCTTGCGCCATTTGTG-3′.

In all cases, samples were tested in triplicate and qPCR reactions were run on a 7500 Real-Time PCR System (Applied Biosystems) following the manufacturer's protocol. The data shown are the mean ± s.e.m. of three experiments, and the relative expression was calculated using the comparative C_t_ method. The qPCR data were analysed by a two-tailed unpaired *t* test.

### Immunofluorescence Staining

Third instar imaginal discs were fixed and stained by standard procedures using the following primary antibodies (dilutions, sources): anti-Eyg (1∶100, [98]), anti-Elav (1∶100, DSHB: Developmental Studies Hybridoma Bank), 4D4 (anti-Wg, 1∶100, DSHB), 4D9 (anti-En, 1∶100, DSHB), anti-phospho-H3 (anti-PH3; 1∶500, Sigma), anti-GFP (1∶1,000, Invitrogen), anti-β-galactosidase (1∶2,000, Cappel), anti-Cut (1∶5,000, DSHB), anti-*D*E-cad (1.50, DSHB), anti-Dac (1∶100, DSHB), anti-Ci (1∶5; a gift from Dr. Holgrem), anti-luciferase (luci27) (1∶200, Thermo Scientific), and anti-DsRed (1∶2,000, Clontech). The secondary antibodies used were conjugated to AlexaFluor-488, -555, -647 (Molecular Probes), and diluted at 1∶400. Discs were mounted in Fluoromount G (Southern Biotechnology), and the images were captured on a Leica TCS-NT Confocal microscope. The RGB Profile Plot function of ImageJ was employed for the intensity profile plots in Figure 6A′ and B″.

### Construction of Sensor Transgenes

The *tub-luc::ihog3′UTR* or *tub-luc::boi3′UTR* constructs were generated by cloning the full-length 3′ UTR of the *Drosophila ihog* or *boi* genes into the 3′ end of the *tub-firefly luciferase* plasmid. To construct the *tub-luc::ihog^mut^3′UTR* reporter, three nucleotides of the predicted binding site for miR-7 in the *ihog* 3′UTR were mutated (AGTCTTCCA to AGTCATGCT) using the QuickChange Site-Directed Mutagenesis kit (Agilent Technologies Inc.). The *tub-eGFP::ihog3′UTR* or *tub-eGFP::boi3′UTR* constructs were generated by cloning the full-length 3′UTR of *ihog* or *boi* genes into the 3′ end of the *tub-eGFP* reporter vector (a gift from Dr. Cohen). The final constructs were verified by sequencing. Transgenic eGFP and luciferase sensor flies were generated on a *w^1118^* background by standard transformation into *Drosophila* embryos (BestGene Inc.).

### Luciferase Reporter Assays

For *Drosophila* S2 cell luciferase assays, cells were co-transfected in 24-well plates as described previously [7] with the *Renilla* luciferase plasmid (75 ng) for normalization and different combinations of the following plasmids: *actin-Gal4* (400 ng), *pUAS-mir-7* or empty *pUAST* (400 ng; [48]), *tub-luc::ihog3′UTR*, *tub-luc::boi3′UTR*, or *tub-luc::ihog^mut^3′UTR* (25 ng). The relative luciferase activity was measured 48 h after transfection using the Dual-Glo Luciferase Reporter Assay system (Promega) according to the manufacturer's instructions. The data shown are the mean ± s.e.m. of three independent experiments, which was analysed by a two-tailed unpaired *t* test.

### Measurement of PH3 Positive Cells

Female virgin *w; ey-Gal4 UAS-Dl/Cy0-GFP* were crossed to males *w; +/+; UAS-DsRed::mir-7* and their F1 progeny larvae (*w; ey-Gal4, UAS-Dl/+; UAS-DsRed::mir-7/+*) were selected by *DsRed* labelling in the pair of eye-antennal discs. The particle analysis function of ImageJ software was used to count PH3-positive nuclei of the confocal images of third instar imaginal discs to generate the data shown in Figure 1J. The analyses of the area of eye disc and antennal disc parts in Figure S4H was done using ImageJ, and data represent mean values of area of eye discs normalized against the antennal disc part in at least six discs *per* genotype.

## Supporting Information

Figure S1 Hh signal along the disc AP axis and Notch-mediated DV growth promoting organizer starts long before the initiation of retinal differentiation. (A) Mid second larval instar (LII) eye disc carrying the enhancer trap line *hh^P30^-lacZ* and stained for ßgalactosidase (hh-Z, green), Wg (red), and Elav (blue). The absence of blue staining denotes that the MF has not yet initiated in this disc. (B) Mid-late LII eye disc carrying the *eyg-lacZ* enhancer trap line and stained for ßgal (blue) and Wg (red). Notch signalling target Eyg expression labels the growth organizer. Disc as in Figure 3F.
(TIF)

Figure S2 The conserved MicroRNA miR-7 and Dl-Notch pathway cooperatively induce eye overgrowth. (A–C) Illustrative images of adult eyes overexpressing *Dl* with the *GS(2)518* line (A) or the *UAS-mir-7* transgene (B–C) with *ey-Gal4*. (A) The overgrown, folded eye tissue often present areas of undifferentiated or poorly differentiated outgrowths (arrowhead) (10%, *n = *200 in A). The undifferentiated outgrowths are seen also in flies co-expressing *Dl* with the *UAS-mir-7* transgene (B and C). (D) Quantification of relative mature *mir-7* RNA levels in larvae carrying *hsp70>mir-7* after heat shock (red bar) or not (blue bar). *P* was calculated using the Student *t* test, and values represented the mean ± sem. of three independent experiments.
(TIF)

Figure S3 Overgrowth and abnormal neuronal differentiation progression in eye discs co-expressing *Dl* and the *GS(2)518* line. Confocal images of eye discs of control wild type (*ey>*, A, C, E, and G) and eye discs overexpressing *Dl* and *mir-7* by *ey-Gal4* (*ey>Dl>GS(2)518*: B, D, F, H–J) and carrying the indicated enhancer trap lines to monitor DV patterning: expression of D marker *mirror-lacZ* (*mirr-Z*), ventral marker *fringe-lacZ* (*fng-Z*), DV organizer-specific marker *Serrate-lacZ* (*Ser-Z*), and *eyegone-lacZ* (*Eq-Z*). Eye discs are stained for ßgalactosidase (green), neuronal marker Elav (blue), or Wg (red). (I–J′) Eye discs are stained for Dac (pink) or *D*E-cadherin (*D*E-cad, green in I and J and grey in I′ and J′) to highlight the morphology of the front of retinal differentiation (MF) and cell shape changes the accompanied neuronal differentiation, respectively. Although it has been postulated that the microRNA mir-7 silences Notch signalling, the overexpression of *mir-7* with *Dl* causes eye disc overgrowth associated with enhanced Dl-Notch signalling as detected by the misexpression of DV organizer-specific markers (F and H). Seldom the pattern of retinal differentiation is highly disrupted in the overgrown discs (F and H) and often the front of neuronal differentiation (arrowhead, I′) is highly irregular or advanced in discs co-expressing *Dl* and *GS(2)518* line. Anterior is to the left. Scale bar, 2 mm.
(TIF)

Figure S4 Overgrowth and abnormal neuronal differentiation progression in eye discs co-expressing *Dl* and the microRNA *mir-7* or the *ihog-IR* or *ci-IR* transgenes. Confocal images of mitotic marker PH3 (blue in A–E; pink in F and green in G), neuronal marker Elav (green, A–F and red in G), and Wg (red, A–D and pink in F) staining of third instar eye-antennal imaginal discs of wild-type *ey-Gal4* (*ey>*, A–A′), *ey-Gal4 UAS-Dl* (*ey>Dl*, B–B′), *ey-Gal4 UAS-Dl/+*; *UAS-mir-7/+* (*ey>Dl>mir-7*, C–D′), *ey-Gal4UAS-Dl/+*; *UAS-ihog-IR/+* (*ey>Dl>ihog-IR*, E–F), and *ey-Gal4 UAS-Dl/+*; *UAS-ci-IR/+* (*ey>Dl>ci-IR*, G). The asterisks point to undifferentiated outgrowth of the eye discs (C, F, and G). Disc in (C) is as in Figure 1H. Note that eye disc overgrowth is also accompanied by advanced or disorganized front of retinal differentiation. The *ey-Gal4* transgene drives expression anterior to the MF (white arrowhead in A), where eye disc cells proliferate asynchronously. Posterior to the MF, subsets of cells start differentiating into photoreceptor neurons visualized by the neuronal marker Elav (green, A) and the remaining cells divide one last time synchronously (row of PH3 cells behind the MF). (H) Quantitation of the eye imaginal disc size of the indicated genotypes. The area for each disc was calculated in pixel using ImageJ and values were normalized with those of the corresponding antennal disc part. As expected, co-expressing *Dl* with the RNAi against *ihog* or *ci* with *ey-Gal4* provoked overgrowth similar, but stronger than the misexpression of the *mir-7*. Anterior is to the left in all images, and dorsal is up.
(TIF)

Figure S5 Quantification of *ihog* and *boi* mRNAs and mature *mir-7* levels. (A) Relative *ihog* mRNA levels in larvae. (B) Relative *boi* mRNA levels in larvae. (C) Relative miR-7 levels in S2 cells transfected with *actGal5* plasmid and with (red bar) or without (blue bar) the *UAS mir-7* plasmid. The values represented the mean ± s.e.m. of at least three independent experiments. Data analysed by a two-tailed unpaired *t* test.
(TIF)

Figure S6 Overexpression of *DsRed::mir-7* by *en-Gal4* in the wing disc also caused reproducible in vivo downregulation of *eGFP* in a *tub-eGFP::ihog-3′UTR* (A) but not in a *tub-eGFP::boi-3′UTR* sensor (B).
(TIF)

Figure S7 Invasive growth caused by co-expressing *Dl* and *ci-IR* in the wing primordium. (A) Wild-type third instar wing imaginal discs. Dpp-GAL4 (*dpp>*) drives expression of *UAS-GFP* (gree) in a narrow band of anterior cells along the AP compartment boundary. Expression of mitotic marker PH3 (blue) and En (red) are also shown. (B) Expression of the RNAi transgene against *ci* (*dpp>ci-IR*) led to anterior expansion of the *dpp* domain visualized by GFP (green) and ectopic P cells (grey in B′) in the A territory at the DV boundary, but the disc is not overgrown. (C, D) Co-expression of *Dl* along with *ci-IR* led to extensive overgrowths. Note that mutant A cells mix with wild-type P (En, positive) cells (arrowheads) in some parts, reminiscent of malignant growth. Expression of Ci (grey in the inset) is also shown in (D).
(TIF)

Figure S8 Blocking Hh signal transduction due to mutations in smoothened enhances organizing activity by Dl-Notch signalling in the mosaic eye and wing discs. (A) Control eye discs carrying MARCM *GFP*(green)-labelled *smo^3^* clones and (B) GFP-labelled clones of *smo^3^* that overexpress *Dl* and stained for *ptc-lacZ* (Ptc-Z, blue) and Ci (blue). Note that the *smo^3^/smo^3^tub-Gal4 UAS-Dl* clones cause nonautonomously advancement of the MF denoted by up-regulated Ci levels, similar to the effect seen in eye discs co-expressing *Dl* with the *mir-7*. (A′) and (B′) show single channel confocal images. (C) Wing discs carrying MARCM *GFP-*labelled clones of *smo^3^* cells and staining for Wg (red, C and C″) and clones of smo3 that overexpress Dl (*smo^3^/smo^3^ tub>Dl*, D–D″). In (D–D″), arrowheads point to ventrally situated clones of anterior origin (visualized by *ptc-lacZ*, not shown). The asterisk points to a clone of ambiguous A origin with weak ectopic Wg only in the anterior portion of the clone. DAPI counterstaining (pink, C″′ and D″′) is shown to illustrate the stimulation of growth of the surrounding tissue by the *smo3 tub-Dl* clones. Genotype in (A and C) is *yw tub-Gal4 UAS-GFP hsp70-Flp; smo^3^ FRT40A ptc-lacZ/tub-Gal80 FRT40A* and in (B and D) is *yw tub-Gal4 UAS-GFP hsp70-Flp; smo^3^ FRT40A ptc-lacZ/tub-Gal80 FRT40A; UAS-Dl/+*.
(TIF)

Figure S9 General genetic scheme of crosses for rescuing experiments in Figure 4. Similar genetic schemes were following the rescue by the UAS-boi transgene in Figure 3J. Larvae carrying both the chromosomes with the transgenes *ey-Gal4 UAS-Dl* (2nd) and *UAS-DsRed::mir-7* (3rd) were selected under a fluorescence binocular (MZFLIII, Leica) for expression of DsRed in the eye under the control of Gal4. The resulting adult males were crossed to female virgins of the genotype *UAS-hh/CyO*. Larvae resulting from the cross were again selected and the DsRed-positive were transferred to a new tube, and the eyes of the resulting non-CyO adults eyes (males and females) were analysed.
(DOCX)

Table S1 Identification of candidate tumour-suppressor gene(s) of *Drosophila* in silico predicted miR-7 target genes in the gain of Dl context.
(DOCX)

Table S2 Direct inhibition by RNAi expression of core Hedgehog pathway genes in the gain of Dl context.
(DOCX)

## Abbreviations

anti-luc: antibody against Luciferase protein
AP: anterior-posterior
Bdx-Gal4: Beadex-Gal4
BL: Bloomington *Drosophila* Stock Center
boi: brother of ihog
ci: cubitus interruptus
Dac: Dacshund
*D*E-cad: *D*E-cadherin
Dl: Delta
DV: dorsal-ventral
eGFP: enhanced Green Fluorescent Protein
Elav: Embryonic lethal abnormal visual system
en-Gal4: engrailed-Gal4
Eq-Z: Equatorial (eyegone)-lacZ
ey-Flp: eyeless-Flippase
eyeless-Gal4: ey-Gal4
fng-Z: fringe-lacZ
FRT: Flippase Recognition Target
Hh: Hedgehog
hsp70-Flp: heat shock promoter 70-Flippase
ihog: interference hedgehog
IR: interference RNA
MF: morphogenetic furrow
mirr-Z: mirror-lacZ
PH3: phopho histone H3
Ptc-Z: Patched-lacZ
qRT-PCR: quantitative reverse transcription polymerase chain reaction
RNAi: RNA interference
s.e.m.: standard error of the mean
Ser-Z: Serrate-lacZ
smo: smoothened
Tub-Gal4: αTubulin84B promoter -Gal4
Tub-Gal80: αTubulin84B promoter -Gal80
UAS: Upstream Activation Sequences
UTR: untraslated region
VDRC: Vienna *Drosophila* RNAi Center
Wg: Wingless
wt: wild type
