# Supplementary figures and images for: Dampening the Signals Transduced through Hedgehog via MicroRNA miR-7 Facilitates Notch-Induced Tumourigenesis
Source: PLoS Biol. 2013 May 7;11(5):e1001554. doi: 10.1371/journal.pbio.1001554 (PMC3646720; doi:10.1371/journal.pbio.1001554)

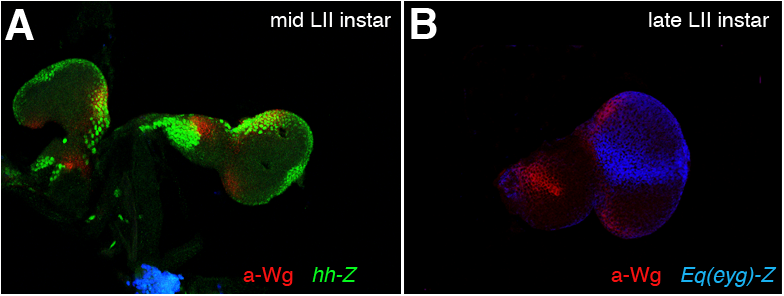

Supplement: Figure S1 — Hh signal along the disc AP axis and Notch-mediated DV growth promoting organizer starts long before the initiation of retinal differentiation. (A) Mid second larval instar (LII) eye disc carrying the enhancer trap line hhP30-lacZ and stained for ßgalactosidase (hh-Z, green), Wg (red), and Elav (blue). The absence of blue staining denotes that the MF has not yet initiated in this disc. (B) Mid-late LII eye disc carrying the eyg-lacZ enhancer trap line and stained for ßgal (blue) and Wg (red). Notch signalling target Eyg expression labels the growth organizer. Disc as in Figure 3F. (TIF) [file pbio.1001554.s001.tif]

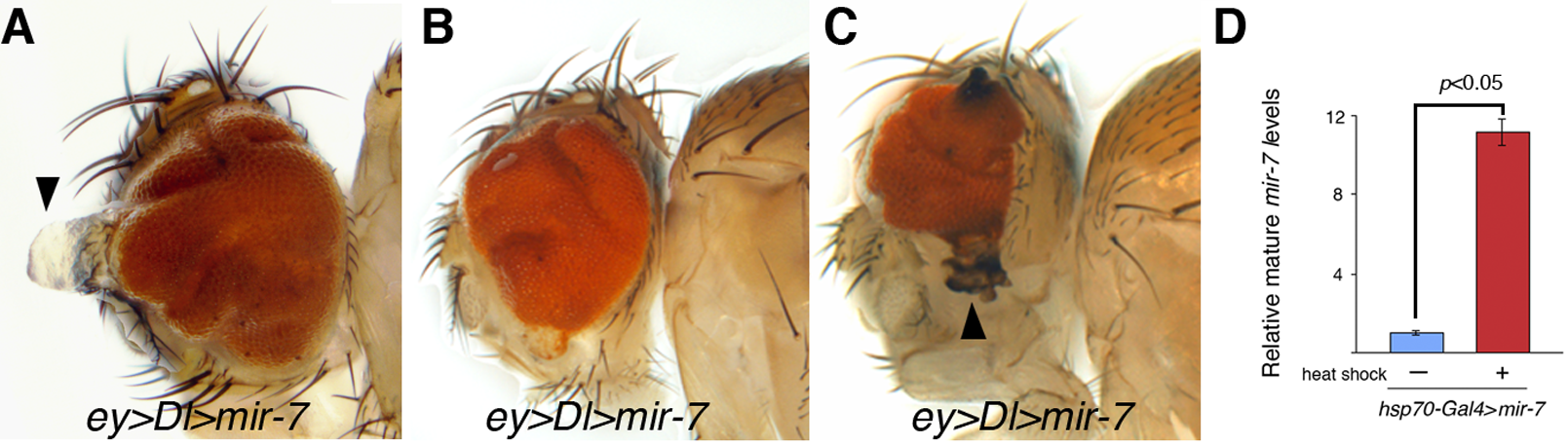

Supplement: Figure S2 — The conserved MicroRNA miR-7 and Dl-Notch pathway cooperatively induce eye overgrowth. (A–C) Illustrative images of adult eyes overexpressing Dl with the GS(2)518 line (A) or the UAS-mir-7 transgene (B–C) with ey-Gal4. (A) The overgrown, folded eye tissue often present areas of undifferentiated or poorly differentiated outgrowths (arrowhead) (10%, n = 200 in A). The undifferentiated outgrowths are seen also in flies co-expressing Dl with the UAS-mir-7 transgene (B and C). (D) Quantification of relative mature mir-7 RNA levels in larvae carrying hsp70>mir-7 after heat shock (red bar) or not (blue bar). P was calculated using the Student t test, and values represented the mean ± sem. of three independent experiments. (TIF) [file pbio.1001554.s002.tif]

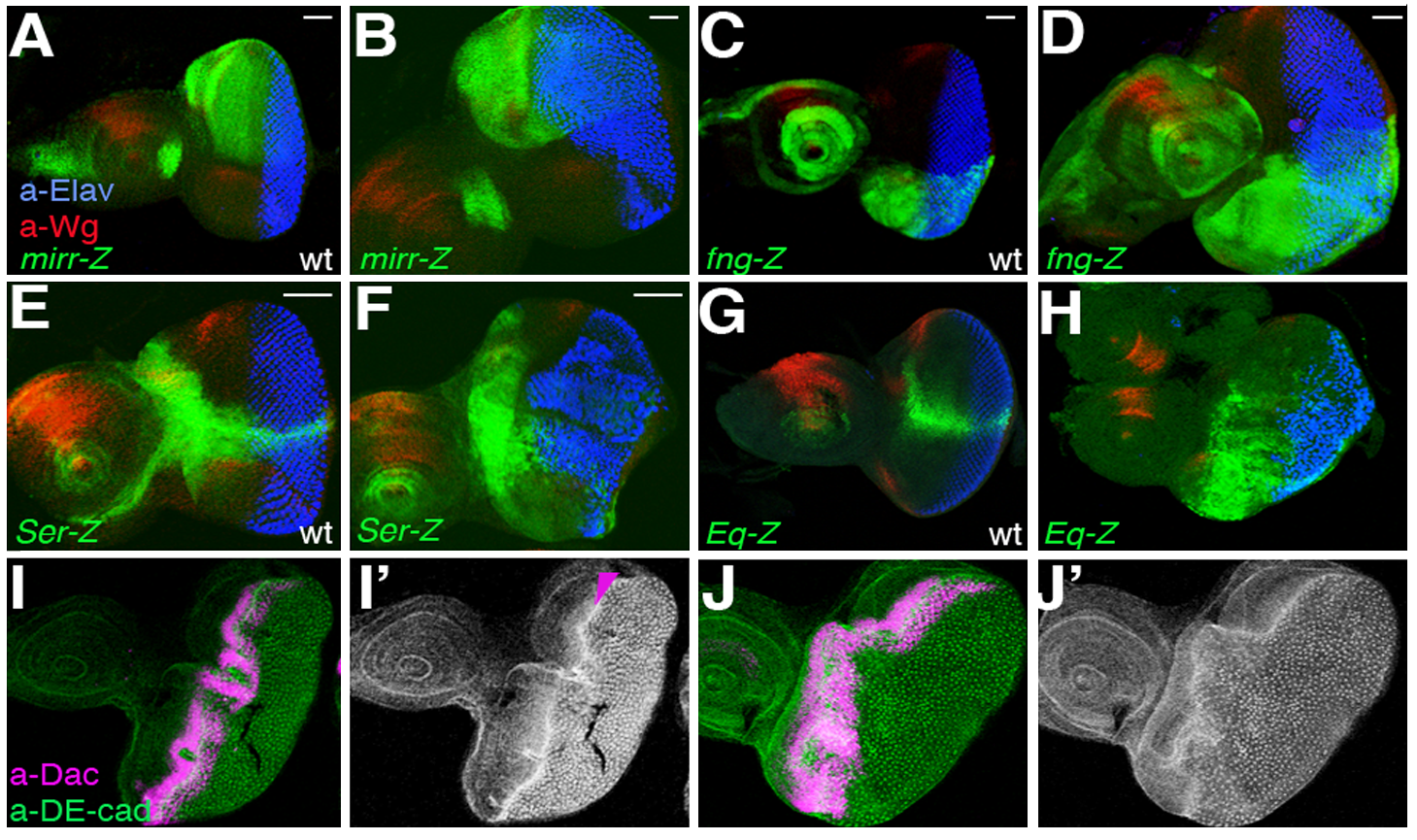

Supplement: Figure S3 — Overgrowth and abnormal neuronal differentiation progression in eye discs co-expressing Dl and the GS(2)518 line. Confocal images of eye discs of control wild type (ey>, A, C, E, and G) and eye discs overexpressing Dl and mir-7 by ey-Gal4 (ey>Dl>GS(2)518: B, D, F, H–J) and carrying the indicated enhancer trap lines to monitor DV patterning: expression of D marker mirror-lacZ (mirr-Z), ventral marker fringe-lacZ (fng-Z), DV organizer-specific marker Serrate-lacZ (Ser-Z), and eyegone-lacZ (Eq-Z). Eye discs are stained for ßgalactosidase (green), neuronal marker Elav (blue), or Wg (red). (I–J′) Eye discs are stained for Dac (pink) or DE-cadherin (DE-cad, green in I and J and grey in I′ and J′) to highlight the morphology of the front of retinal differentiation (MF) and cell shape changes the accompanied neuronal differentiation, respectively. Although it has been postulated that the microRNA mir-7 silences Notch signalling, the overexpression of mir-7 with Dl causes eye disc overgrowth associated with enhanced Dl-Notch signalling as detected by the misexpression of DV organizer-specific markers (F and H). Seldom the pattern of retinal differentiation is highly disrupted in the overgrown discs (F and H) and often the front of neuronal differentiation (arrowhead, I′) is highly irregular or advanced in discs co-expressing Dl and GS(2)518 line. Anterior is to the left. Scale bar, 2 mm. (TIF) [file pbio.1001554.s003.tif]

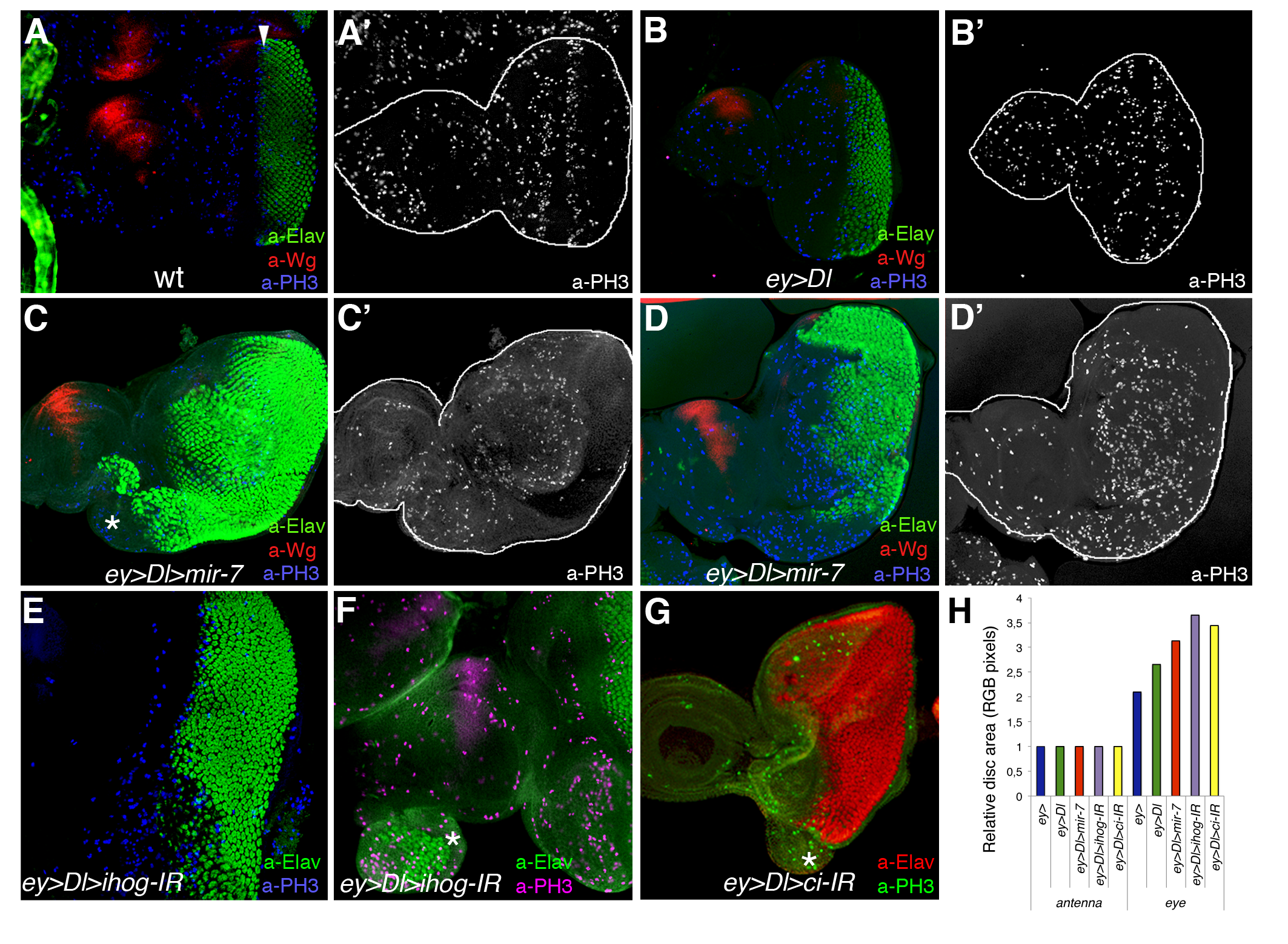

Supplement: Figure S4 — Overgrowth and abnormal neuronal differentiation progression in eye discs co-expressing Dl and the microRNA mir-7 or the ihog-IR or ci-IR transgenes. Confocal images of mitotic marker PH3 (blue in A–E; pink in F and green in G), neuronal marker Elav (green, A–F and red in G), and Wg (red, A–D and pink in F) staining of third instar eye-antennal imaginal discs of wild-type ey-Gal4 (ey>, A–A′), ey-Gal4 UAS-Dl (ey>Dl, B–B′), ey-Gal4 UAS-Dl/+; UAS-mir-7/+ (ey>Dl>mir-7, C–D′), ey-Gal4UAS-Dl/+; UAS-ihog-IR/+ (ey>Dl>ihog-IR, E–F), and ey-Gal4 UAS-Dl/+; UAS-ci-IR/+ (ey>Dl>ci-IR, G). The asterisks point to undifferentiated outgrowth of the eye discs (C, F, and G). Disc in (C) is as in Figure 1H. Note that eye disc overgrowth is also accompanied by advanced or disorganized front of retinal differentiation. The ey-Gal4 transgene drives expression anterior to the MF (white arrowhead in A), where eye disc cells proliferate asynchronously. Posterior to the MF, subsets of cells start differentiating into photoreceptor neurons visualized by the neuronal marker Elav (green, A) and the remaining cells divide one last time synchronously (row of PH3 cells behind the MF). (H) Quantitation of the eye imaginal disc size of the indicated genotypes. The area for each disc was calculated in pixel using ImageJ and values were normalized with those of the corresponding antennal disc part. As expected, co-expressing Dl with the RNAi against ihog or ci with ey-Gal4 provoked overgrowth similar, but stronger than the misexpression of the mir-7. Anterior is to the left in all images, and dorsal is up. (TIF) [file pbio.1001554.s004.tif]

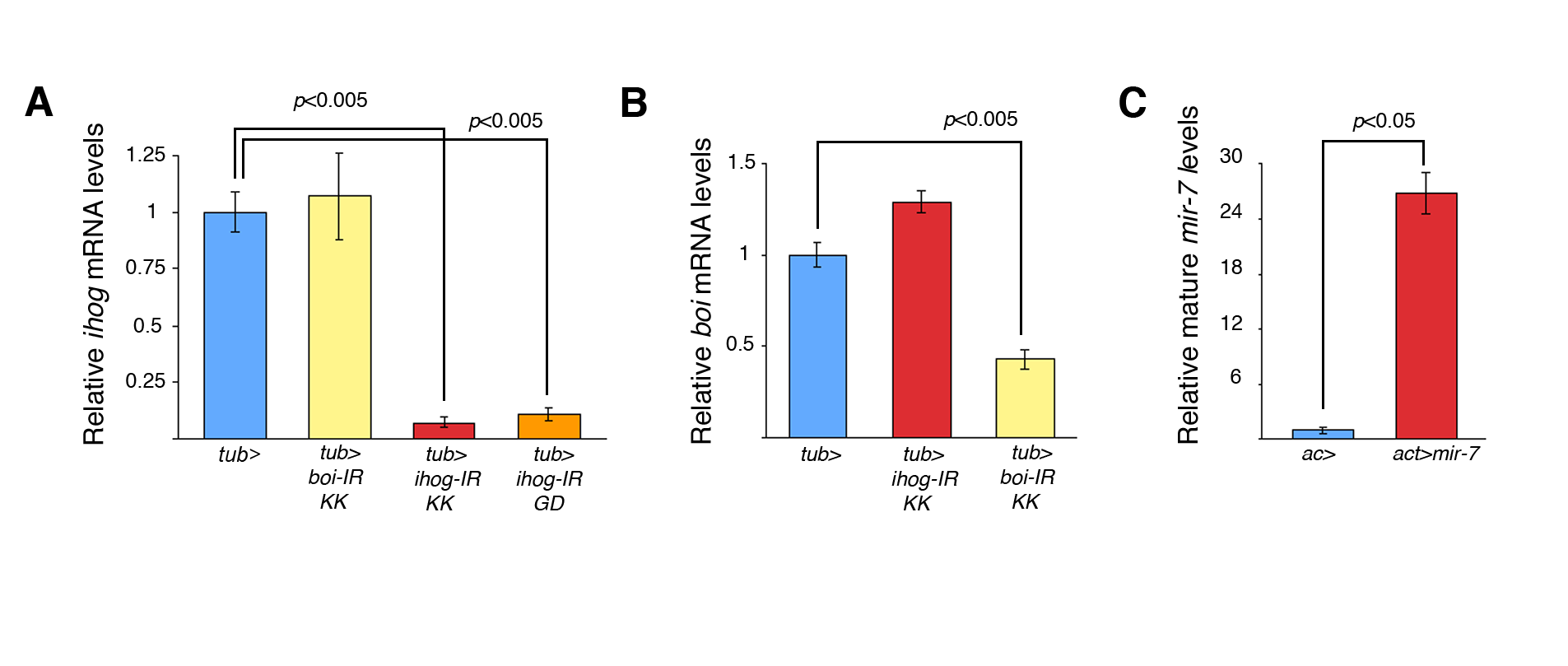

Supplement: Figure S5 — Quantification of ihog and boi mRNAs and mature mir-7 levels. (A) Relative ihog mRNA levels in larvae. (B) Relative boi mRNA levels in larvae. (C) Relative miR-7 levels in S2 cells transfected with actGal5 plasmid and with (red bar) or without (blue bar) the UAS mir-7 plasmid. The values represented the mean ± s.e.m. of at least three independent experiments. Data analysed by a two-tailed unpaired t test. (TIF) [file pbio.1001554.s005.tif]

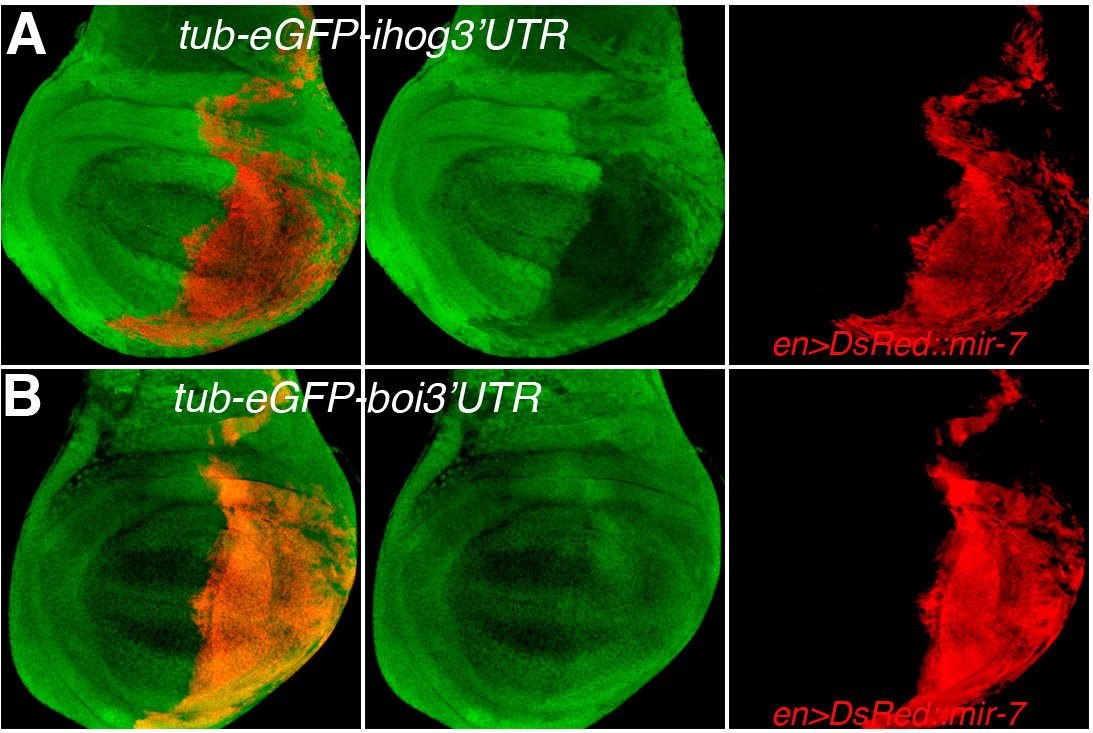

Supplement: Figure S6 — Overexpression of DsRed::mir-7 by en-Gal4 in the wing disc also caused reproducible in vivo downregulation of eGFP in a tub-eGFP::ihog-3′UTR (A) but not in a tub-eGFP::boi-3′UTR sensor (B). (TIF) [file pbio.1001554.s006.tif]

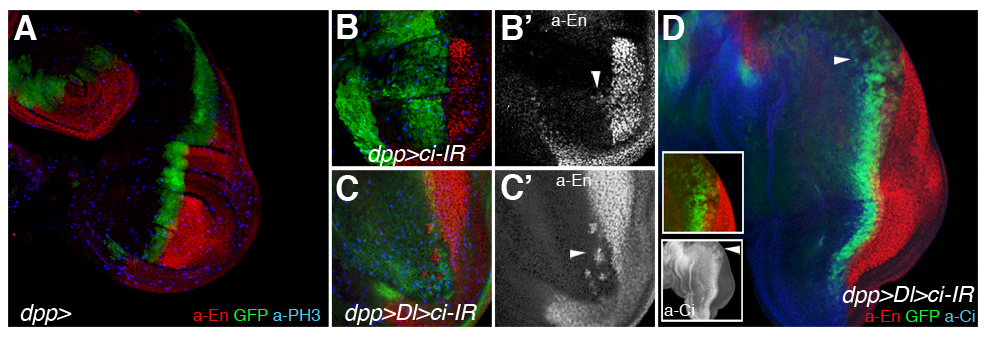

Supplement: Figure S7 — Invasive growth caused by co-expressing Dl and ci-IR in the wing primordium. (A) Wild-type third instar wing imaginal discs. Dpp-GAL4 (dpp>) drives expression of UAS-GFP (gree) in a narrow band of anterior cells along the AP compartment boundary. Expression of mitotic marker PH3 (blue) and En (red) are also shown. (B) Expression of the RNAi transgene against ci (dpp>ci-IR) led to anterior expansion of the dpp domain visualized by GFP (green) and ectopic P cells (grey in B′) in the A territory at the DV boundary, but the disc is not overgrown. (C, D) Co-expression of Dl along with ci-IR led to extensive overgrowths. Note that mutant A cells mix with wild-type P (En, positive) cells (arrowheads) in some parts, reminiscent of malignant growth. Expression of Ci (grey in the inset) is also shown in (D). (TIF) [file pbio.1001554.s007.tif]

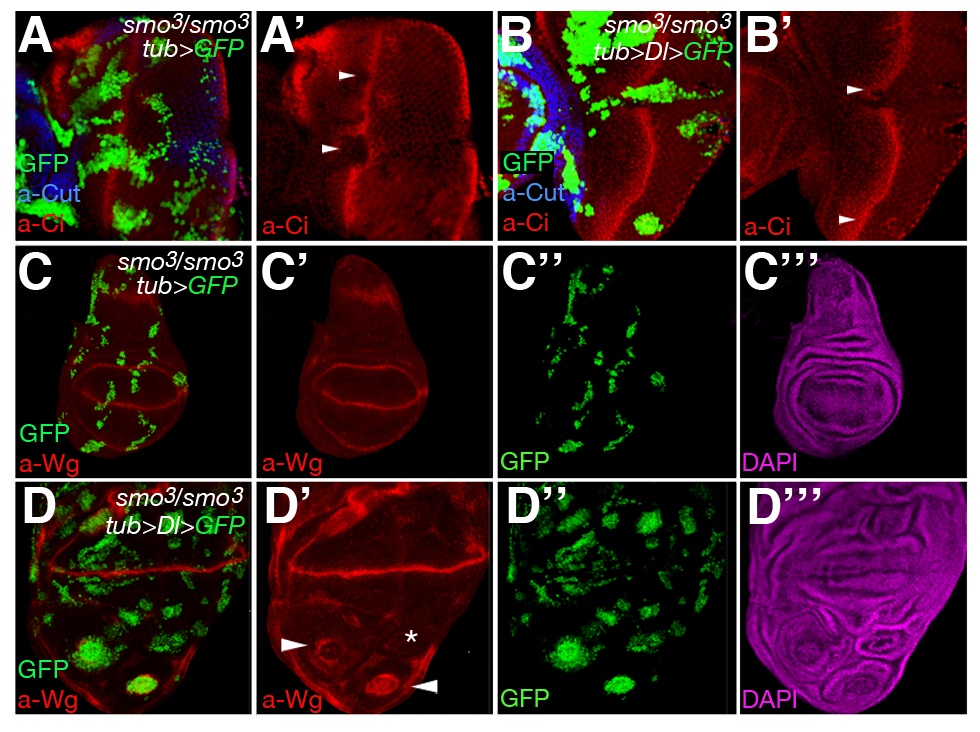

Supplement: Figure S8 — Blocking Hh signal transduction due to mutations in smoothened enhances organizing activity by Dl-Notch signalling in the mosaic eye and wing discs. (A) Control eye discs carrying MARCM GFP(green)-labelled smo3 clones and (B) GFP-labelled clones of smo3 that overexpress Dl and stained for ptc-lacZ (Ptc-Z, blue) and Ci (blue). Note that the smo3/smo3tub-Gal4 UAS-Dl clones cause nonautonomously advancement of the MF denoted by up-regulated Ci levels, similar to the effect seen in eye discs co-expressing Dl with the mir-7. (A′) and (B′) show single channel confocal images. (C) Wing discs carrying MARCM GFP-labelled clones of smo3 cells and staining for Wg (red, C and C″) and clones of smo3 that overexpress Dl (smo3/smo3 tub>Dl, D–D″). In (D–D″), arrowheads point to ventrally situated clones of anterior origin (visualized by ptc-lacZ, not shown). The asterisk points to a clone of ambiguous A origin with weak ectopic Wg only in the anterior portion of the clone. DAPI counterstaining (pink, C″′ and D″′) is shown to illustrate the stimulation of growth of the surrounding tissue by the smo3 tub-Dl clones. Genotype in (A and C) is yw tub-Gal4 UAS-GFP hsp70-Flp; smo3 FRT40A ptc-lacZ/tub-Gal80 FRT40A and in (B and D) is yw tub-Gal4 UAS-GFP hsp70-Flp; smo3 FRT40A ptc-lacZ/tub-Gal80 FRT40A; UAS-Dl/+. (TIF) [file pbio.1001554.s008.tif]
