# Supplementary material for: Dampening the Signals Transduced through Hedgehog via MicroRNA miR-7 Facilitates Notch-Induced Tumourigenesis
Source: PLoS Biol. 2013 May 7;11(5):e1001554. doi: 10.1371/journal.pbio.1001554 (PMC3646720; doi:10.1371/journal.pbio.1001554)
Supplement: Figure S9 — General genetic scheme of crosses for rescuing experiments in Figure 4. Similar genetic schemes were following the rescue by the UAS-boi transgene in Figure 3J. Larvae carrying both the chromosomes with the transgenes ey-Gal4 UAS-Dl (2nd) and UAS-DsRed::mir-7 (3rd) were selected under a fluorescence binocular (MZFLIII, Leica) for expression of DsRed in the eye under the control of Gal4. The resulting adult males were crossed to female virgins of the genotype UAS-hh/CyO. Larvae resulting from the cross were again selected and the DsRed-positive were transferred to a new tube, and the eyes of the resulting non-CyO adults eyes (males and females) were analysed. (DOCX) [file pbio.1001554.s009.docx]

**Supplementary Figure S9**
