# Supplementary material for: Dampening the Signals Transduced through Hedgehog via MicroRNA miR-7 Facilitates Notch-Induced Tumourigenesis
Source: PLoS Biol. 2013 May 7;11(5):e1001554. doi: 10.1371/journal.pbio.1001554 (PMC3646720; doi:10.1371/journal.pbio.1001554)
Supplement: Table S1 — Identification of candidate tumour-suppressor gene(s) of Drosophila in silico predicted miR-7 target genes in the gain of Dl context. (DOCX) [file pbio.1001554.s010.docx]

**Supplementary Table S1.** Identification of Candidate Tumour-Suppressor Gene(s) of*Drosophila in silico* Predicted miR-7 Target Genes in the Gain of Dl Context**.**

| **Gene^a^** | **RNAi** | **Stock** | **FlyBase ID** | **FlyBase Genotype** | **Phenotype ^c^** | **Incidence** |
| --- | --- | --- | --- | --- | --- | --- |
|  | **Collection**^b^ | **Number** |  |  | ***ey-Gal4>Dl>dcr2*** | **(n>50) ^d^** |
| *5-HT1β* | VDRC | 46485 | FBst0466727 | w^1118^; P{GD17040}v46485/TM3 | N.C. |  |
| *5-HT1β* | BL | 25833 | FBst0025833 | y^1^ v^1^; P{y[+t7.7]v[+t1.8]=TRiP.JF01851}attP2 | N.C. |  |
| *aop* | BL | 34909 | FBst0034909 | y^1^ sc* v^1^; P{TRiP.HMS01256}attP2 | Early lethal | 100% |
| *aop* | BL | 26759 | FBst0026759 | y^1^ v^1^; P{y[+t7.7v[+t1.8]=TRiP.JF02323}attP2/TM3 | N.C. |  |
| *ato* | BL | 26316 | FBst0026316 | y^1^ v^1^;; P{TRiP.JF02089}attP2 | Hypo | 100% |
| *BobA* | VDRC | 29796 | FBst0458154 | w^1118^; P{GD15234}v29796 | N.C. |  |
| *bowl* | BL | 27074 | FBst0027074 | y^1^ v^1^; P{TRiP.JF02419}attP2 | Hyper | 10% |
| *bowl* | VDRC | v102050 | FBst0473922 | P{KK110520}VIE-260B | T | 70% |
| *Cad87A* | BL | 28716 | FBst0028716 | y^1^ v^1^; P{TRiP.JF03143}attP2 | N.C. |  |
| *Cad87A* | VDRC | 105901 | FBst0477727 | P{KK104234}VIE-260B | N.C. |  |
| *Cad87A* | VDRC | 8578 | FBst0471146 | w^1118^; P{GD3637}v8578 | Hyper | 40% |
| *CG10444* | VDRC | 4722 | FBst0467144 | w^1118^; P{GD2104}v4722 | N.C. |  |
| *CG10444* | VDRC | 107008 | FBst0478831 | P{KK101545}VIE-260B | N.C. |  |
| *CG11319* | VDRC | 7621 | FBst0470766 | w^1118^; P{GD954}v7621 | N.C. |  |
| *CG12488* | VDRC | 105028 | FBst0476856 | P{KK113020}VIE-260B | N.C. |  |
| *CG13908* | BL | 28645 | FBst0028645 | y^1^ v^1^; P{TRiP.JF03060}attP2 | N.C. |  |
| *CG13908* | VDRC | 47179 | FBst0467118 | w^1118^; P{GD16461}v47179 | N.C. |  |
| *CG18549* | VDRC | 107272 | FBst0479094 | P{KK102196}VIE-260B | N.C. |  |
| *CG31472* | VDRC | 105941 | FBst0477767 | P{KK107976}VIE-260B | N.C. |  |
| *CG32103* | VDRC | 108078 | FBst0479890 | P{KK100089}VIE-260B | N.C. |  |
| *CG7272* | VDRC | 8375 | FBst0471046 | w^1118^; P{GD2471}v8375 | N.C. |  |
| *CG9368* | BL | 28292 | FBst0028292 | y^1^ v^1^; P{TRiP.JF02922}attP2 | N.C. |  |
| *CG9368* | VDRC | 29786 | FBst0458148 | w^1118^; P{GD15225}v29786/CyO | Hyper | 60% |
| *cpa* | VDRC | 100773 | FBst0472646 | P{KK108554}VIE-260B | Hypo | 100% |
| *da* | BL | 29326 | FBst0029326 | y^1^ v^1^; P{TRiP.JF02488}attP2 | Hypo | 100% |
| *da* | VDRC | 105258 | FBst0477086 | P{KK104800}VIE-260B | Hypo | 100% |
| *dachs* | BL | 27664 | FBst0027664 | y^1^ v^1^; P{TRiP.JF02743}attP2 | N.C. |  |
| *dachs* | VDRC | 102550 | FBst0474419 | P{KK111964}VIE-260B | N.C. |  |
| *da* | BL | 26319 | FBst0026319 | y^1^ v^1^; P{TRiP.JF02092}attP2 | Hypo | 100% |
| *gho* | VDRC | 106929 | FBst0478752 | P{KK102658}VIE-260B | Pupal lethal | 100% |
| *h* | BL | 27738 | FBtp0052562 | y^1^ v^1^; P{TRiP.JF02822}attP2 | T | 100% |
| *HLHm3* | BL | 25977 | FBst0025977 | y^1^ v^1^; P{TRiP.JF01999}attP2 | N.C. |  |
| *HLHm4* | BL | 29378 | FBst0029378 | y^1^ v^1^; P{TRiP.JF03310}attP2 | N.C. |  |
| *HLHm5* | BL | 26201 | FBst0026201 | y^1^ v^1^; P{TRiP.JF02099}attP2 | N.C. |  |
| *HLHm5* | VDRC | 47124 | FBst0467079 | w^1118^; P{GD16281}v47124 | N.C. |  |
| *HLHm5* | VDRC | 101948 | FBst0473820 | P{KK110311}VIE-260B | N.C. |  |
| *HLHmγ* | BL | 25978 | FBst0025978 | y^1^ v^1^; P{TRiP.JF02000}attP2 | N.C. |  |
| *HLHmδ* | VDRC | v13077 | FBst0450833 | w^1118^; P{GD4458}v13077 | Hypo | 100% |
| *HLHmδ* | VDRC | v100056 | FBst0471930 | P{KK103234}VIE-260B | T | 30% |
| *ihog* | VDRC | v29897 | FBst0458215 | w^1118^; P{GD14317}v29897 | T | 80% |
| *ihog* | VDRC | v102602 | FBst0474471 | P{KK112149}VIE-260B | T | 100% |
| *jbug* | VDRC | 102221 | FBst0474090 | P{KK111138}VIE-260B | N.C. |  |
| *jbug* | VDRC | 28471 | FBst0457492 | w^1118^; P{GD13033}v28471 | N.C. |  |
| *Lama* | VDRC | 31312 | FBst0031312 | y^1^ v^1^; P{TRiP.JF01259}attP2 | Hyper | 100% |
| *mbc* | VDRC | 16044 | FBst0452122 | w^1118^; P{GD6965}v16044 | N.C. |  |
| *Rac1* | BL | 28985 | FBst0028985 | y^1^ v^1^; P{TRiP.JF02813}attP2 e* | N.C. |  |
| *Ssadh* | VDRC | 106637 | FBst0472903 | P{KK106637}VIE-260B | N.C. |  |
| *Sucb* | VDRC | 101554 | FBst0473427 | P{KK109063}VIE-260B | N.C. |  |
| *Teh1* | VDRC | 46364 | FBst0466655 | w^1118^; P{GD2891}v46364 | N.C. |  |
| *Teh1* | VDRC | 102816 | FBst0474681 | P{KK103880}VIE-260B | N.C. |  |
| *Tom* | VDRC | v101652 | FBst0473525 | P{KK105340}VIE-260B | N.C. |  |
| *Tom* | VDRC | v36614 | FBst0461768 | w^1118^; P{GD14880}v36614 | T | 40% |

^a^ Genes sorted by alphabetic order. Note that generally the KK lines provoke stronger phenotypes than GD lines and that some RNAi lines targeting the same gene caused inconsistent phenotype.

^b^ VDRC: Vienna Drosophila RNAi Center (http://stockcenter.vdrc.at/control/main), BL: Bloomington Drosophila Stock Center (http://fly.bio.indiana.edu/)

^c^ T: Tumor; Hyper: Hyperplasia; Hypo: Hypoplasia, NC: No Change. Phenotype obtained from the cross between males from the different RNAi lines and females *ey>Dl>dcr2.*

^d^ *n* = total number of eyes counted.

The list above contains genes that are predicted to be target for *D. melanogaster* miR-7 by the following databases: CBIO (http://cbio.mskcc.org/mirnaviewer/), TargetScan (http://www.targetscan.org/fly_11/), PicTar (http:// http://pictar.mdc-berlin.de/cgi-bin/PicTar_fly.cgi?species=fly/) and MirBase (http://microrna.sanger.ac.uk/sequences/). In addition, the highlighted rows correspond to genes that have been already validated as miR-7 targets in other cellular contexts (Li *et al.*, 2009 and citations therein).
