# Supplementary material for: Dampening the Signals Transduced through Hedgehog via MicroRNA miR-7 Facilitates Notch-Induced Tumourigenesis
Source: PLoS Biol. 2013 May 7;11(5):e1001554. doi: 10.1371/journal.pbio.1001554 (PMC3646720; doi:10.1371/journal.pbio.1001554)
Supplement: Table S2 — Direct inhibition by RNAi expression of core Hedgehog pathway genes in the gain of Dl context. (DOCX) [file pbio.1001554.s011.docx]

**Supplementary Table S2.** Direct Inhibition by RNAi Expression of Core Hedgehog Pathway Genes in the Gain of Dl Context.

| **Gene ^a^** | **RNAi** | **Stock** | **FlyBase ID** | **FlyBase Genotype** | **Phenotype ^c^** | **Incidence** |
| --- | --- | --- | --- | --- | --- | --- |
|  | **Collection**^b^ | **Number** |  |  | ***ey-Gal4>Dl>dcr2*** | **(*n*>200)^d^** |
| *boi* | VDRC | 108265 | FBst0480077 | P{KK103113}VIE-260B | N.C. |  |
| *ci* | VDRC | 51479 | FBst0020742 | w^1118^; P{GD1403}v51479 | Hyper | 100% |
| *ci* | VDRC | 105620 | FBst0026821 | P{KK100760}VIE-260B | T | 100% |
| *ci* | BL | 28984 | FBst0032272 | y^1^ v^1^; P{TRiP.JF01715}attP2 | Met | 10% |
| *hh* | BL | 25794 | FBst0025794 | y^1^ v^1^; P{TRiP.JF01804}attP2 | T | 100% |
| *hh* | VDRC | 1402 | FBst0451269 | w^1118^; P{GD193}v1402 | T | 30% |
| *smo* | BL | 27037 | FBst0027037 | y^1^ v^1^; P{TRiP.JF02363}attP2 | T | 78% |
| *smo* | VDRC | 9542 | FBst0471542 | w^1118^; P{GD577}v9542 | T | 80% |
| *pka-C1* | VDRC | 101524 | FBst0473397 | P{KK108966}VIE-260B | Hypoplasia | 100% |

^a^ Canonical positive (*boi, ci, hh, smo*) and negative (*pka-C1*) Hh pathway genes sorted by alphabetic order. Note that generally the KK lines provoke stronger phenotypes than GD lines.

^b^ VDRC: Vienna Drosophila RNAi Center (http://stockcenter.vdrc.at/control/main), BL: Bloomington Drosophila Stock Center (http://fly.bio.indiana.edu/)

^c^ Met: Metastasis; T: Tumor; Hyper: Hyperplasia; NC: No Change. Phenotype obtained from the cross between males from the different RNAi lines and females *ey>Dl>dcr2.*

^d^*n* = total number of eyes counted.
